# Retinol-Binding Protein 4 Accelerates Metastatic Spread and Increases Impairment of Blood Flow in Mouse Mammary Gland Tumors

**DOI:** 10.3390/cancers12030623

**Published:** 2020-03-07

**Authors:** Diana Papiernik, Anna Urbaniak, Dagmara Kłopotowska, Anna Nasulewicz-Goldeman, Marcin Ekiert, Marcin Nowak, Joanna Jarosz, Monika Cuprych, Aleksandra Strzykalska, Maciej Ugorski, Rafał Matkowski, Joanna Wietrzyk

**Affiliations:** 1 Department of Experimental Oncology, Hirszfeld Institute of Immunology and Experimental Therapy, Polish Academy of Sciences, 53-114 Wroclaw, Poland; diana.papiernik@gmail.com (D.P.); anna.urbaniak@hirszfeld.pl (A.U.); dagmara.klopotowska@hirszfeld.pl (D.K.); joanna.jarosz@hirszfeld.pl (J.J.); monika.cuprych@hirszfeld.pl (M.C.); s.aleksandra0@wp.pl (A.S.); 2 Division of Surgical Oncology and Clinical Oncology, Department of Oncology, Wroclaw Medical University, 50-367 Wroclaw, Poland; marcin.ekiert@umed.wroc.pl (M.E.); rafal.matkowski@umed.wroc.pl (R.M.); 3 Wroclaw Comprehensive Cancer Center, 53-413 Wroclaw, Poland; 4 Faculty of Veterinary Medicine, Wroclaw University of Environmental and Life Sciences, 50-375 Wroclaw, Poland; marcin.nowak@upwr.edu.pl (M.N.); maciej.ugorski@upwr.edu.pl (M.U.)

**Keywords:** RBP4, metastasis, breast cancer, angiogenesis, endothelial dysfunction, STAT3, VEGF, endothelin-1

## Abstract

Retinol-binding protein 4 (RBP4) is proposed as an adipokine that links obesity and cancer. We analyzed the role of RBP4 in metastasis of breast cancer in patients and in mice bearing metastatic 4T1 and nonmetastatic 67NR mammary gland cancer. We compared the metastatic and angiogenic potential of these cells transduced with *Rbp4* (4T1/RBP4 and 67NR/RBP4 cell lines). Higher plasma levels of RBP4 were observed in breast cancer patients with metastatic tumors than in healthy donors and patients with nonmetastatic cancer. Increased levels of RBP4 were observed in plasma, tumor tissue, liver, and abdominal fat. Moreover, the blood vessel network was highly impaired in mice bearing 4T1 as compared to 67NR tumors. RBP4 transductants showed further impairment of blood flow and increased metastatic potential. Exogenous RBP4 increased lung settlement by 67NR and 4T1 cells. In vitro studies showed increased invasive and clonogenic potential of cancer cells treated with or overexpressing RBP4. This effect is not dependent on STAT3 phosphorylation. RBP4 enhances the metastatic potential of breast cancer tumors through a direct effect on cancer cells and through increased endothelial dysfunction and impairment of blood vessels within the tumor.

## 1. Introduction

Recent studies utilizing the model of mouse mammary gland cancer 4T1, reflecting a basal-like phenotype (in human: negative for nuclear estrogen receptor (ER) α, progesterone receptor (PR) and human epidermal growth factor receptor 2 (HER2), i.e., triple-negative, and positive for epidermal growth factor receptor (EGFR)) [1,2,3,4], have shown a predominant role of endothelium damage during the metastatic process of these cells [5,6,7]. For instance, it is evidenced that endothelial dysfunction in the lungs, which was assessed as decreased activity and phosphorylation of endothelial nitric oxide synthase (eNOS) resulting in a low nitric oxide (NO) production state, was an early event in breast cancer pulmonary metastasis. These processes precede the onset of a phenotypic switch in the lung endothelium toward a mesenchymal phenotype (EndMT), which is parallel to the appearance of the first pulmonary metastatic colonies [7]. Therefore, therapeutic strategies that aim to normalize endothelial dysfunction can decrease the metastatic potential of this type of breast cancer [8,9,10].

Apart from its involvement in cancer development, endothelial dysfunction plays an important role in the development of cardiovascular diseases and atherosclerosis. Moreover, in type 2 diabetes mellitus, endothelial dysfunction and insulin resistance often coexist at the earliest stage of atherosclerosis with elevation of serum retinol-binding protein 4 (RBP4), a specific retinol transporter in the blood [11]. It is documented that RBP4 induces inflammation of endothelial cells in vitro. This action is due to the stimulation of proinflammatory molecules involved in leukocyte recruitment and their adherence to endothelium, and it is independent of retinol and the RBP4 membrane receptor STRA6 [12]. Endothelial inflammation induced by RBP4 is largely mediated by toll-like receptor 4 (TLR4), and in part, through the c-Jun N-terminal kinase (JNK) and p38 mitogen-activated protein kinase (MAPK) signaling pathways [13]. Moreover, in isolated aorta rings, RBP4 treatment significantly increased NO production, stimulating the PI3K/Akt/eNOS pathway [14].

RBP4, classified as adipokine [15], is proposed as the protein linking obesity and cancer [16]. Studies have shown various correlations between RBP4 plasma/tumor tissue levels and the development of certain types of cancer. For instance, Fei et al. reported lower RBP4 serum levels in patients with colon cancer than in healthy individuals [17]; on the other hand, Karunanithi et al. and Abola et al. have shown that elevated RBP4 is associated with colon cancer progression and liver metastasis [18,19]. There are also studies showing the importance of this protein in ovarian, renal, hepatocellular, oral squamous cell, and pancreatic cancer patients [20,21,22,23,24]. Such analyses conducted in patients with breast cancer suggested a link between elevated RBP4 and the risk of breast cancer [25]. The proposed mechanisms of RBP4 effects on cancer cells are dependent on the activation of the signaling receptor and transporter of retinol STRA6 by bound RBP4 and further transduction of the JAK2-STAT3 signaling cascade [18]. Other authors have shown that knockdown of RBP4 significantly reduces ovarian cancer cell migration and proliferation driven through the RhoA/Rock1 and extracellular signal-regulated kinase (ERK) pathways [26].

The aim of our studies was to explain the role of RBP4 protein in the growth and metastatic spread of two murine breast cancer isogenic cell lines (derived from a single tumor of BALB/c mouse); metastatic 4T1 and nonmetastatic 67NR, representing basal-like and luminal-like phenotype, respectively [2,4]. Moreover, because, in many cases, cancer is associated with the aging process (largely reviewed in [27]) and aging affects the metastatic phenotype of cancer cells as well as tumor angiogenesis [28,29,30], we decided to include in our studies young and aged animals. To the best of our knowledge, no previous studies have investigated the effect of RBP4 protein on the metastatic spread of cancer.

## 2. Results

### 2.1. Impaired Angiogenesis in 4T1 Metastatic Tumors Compared to That in Nonmetastatic 67NR Tumors

**Figure 1:**
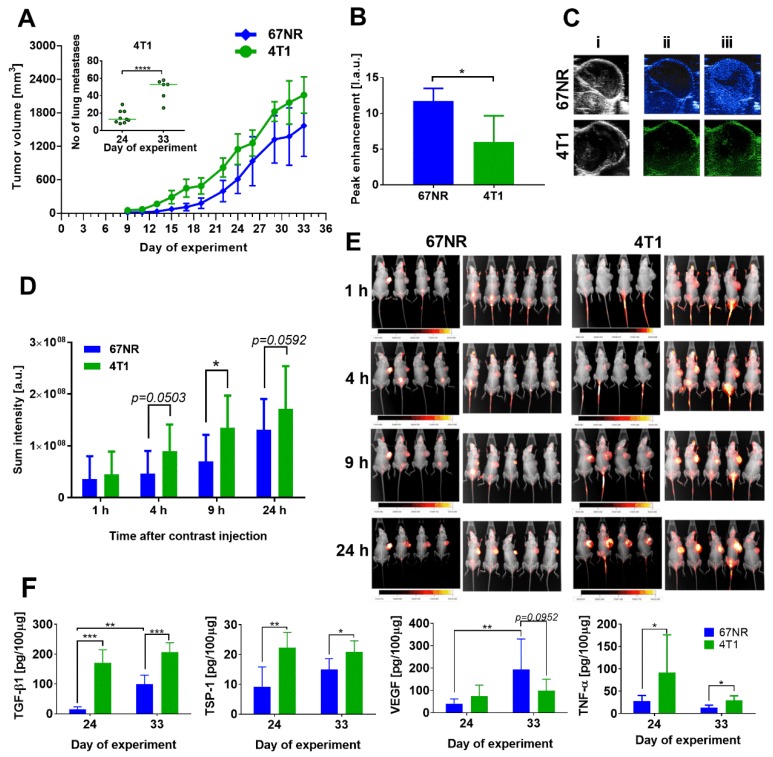
Basic characteristics of 67NR tumors compared to 4T1 mouse mammary gland tumors in 6- to 8-week-old mice. (**A**) Tumor growth kinetics of 67NR and 4T1 cancer and in inserted graph: number of metastases on 2 steps of 4T1 tumor progression. (**B**) Analysis of tumor blood perfusion: peak enhancement parameter. (**C**) Representative pictures of (**i**) Ultrasonography (USG) image, (**ii**) tumors before contrast agent injection, (**iii**) tumors maximally filled with the contrast agent. (**D**) Blood vessel permeability: fluorescence of IRDye^®^ 800CW fluorescent dye PEG Contrast Agent. (**E**) Pictures of X-ray and fluorescence images of mice. (**F**) Tumor tissue level of transforming growth factor β1 (TGF-β1), thrombospondin 1 (TSP-1), vascular endothelial growth factor (VEGF), and tumor necrosis factor α (TNF-α). Data presented as mean ± SD or data for individual mice (insert in figure **A**). Number of mice per group: (**A**) 6–9; (**B**) 3–4; (**D**) 9; (**F**) 5. Statistical analysis: Tukey’s multiple comparison test * *p* < 0.05, ** *p* < 0.01, *** *p* < 0.001, **** *p* < 0.0001.

The 4T1 and 67NR cells from in vitro culture were injected orthotopically (ort.) into the mammary fat pad of 6–8-week-old mice. The mice were observed and tumor growth was measured. Kinetics of growth of 4T1 and 67NR tumors were similar, but only 4T1 cells formed lung metastatic foci (Figure 1A).

**Figure 2:**
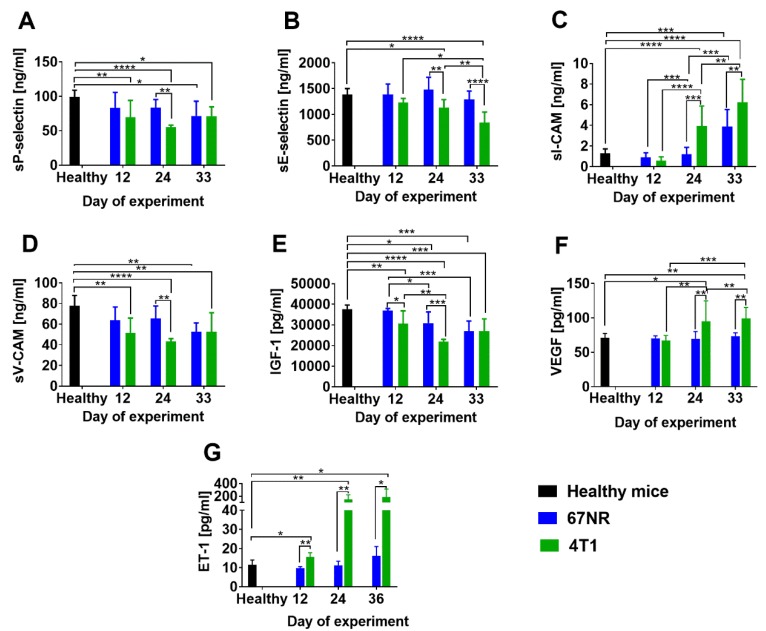
Plasma level of soluble proteins in 6- to 8-week-old mice bearing 67NR tumors compared to 4T1 mouse mammary gland tumors. (**A**) sP- and (**B**) sE-selectin, (**C**) intercellular adhesion molecule (sI-CAM) and (**D**) vascular cell adhesion molecule 1 (sV-CAM), (**E**) insulin-like growth factor 1 (IGF-1), (**F**) vascular endothelial growth factor (VEGF), and (**G**) endothelin-1 (ET-1). Data presented as mean ± SD. Number of mice per group: 5. Statistical analysis: Tukey’s multiple comparison test * *p* < 0.05, ** *p* < 0.01, *** *p* < 0.001, **** *p* < 0.0001.

Literature data [4] and our histopathological analyses confirmed that no cancer cells were detected in the lungs of 67NR tumor-bearing mice (Figure S1A). The 4T1 tumors implanted ort. demonstrated decreased blood flow (Figure 1B,C) and increased blood vessel permeability as compared to 67NR tumors (Figure 1D,E). Tumor tissue levels of transforming growth factor β1 (TGF-β1), thrombospondin 1 (TSP-1), and tumor necrosis factor α (TNF-α) were higher in 4T1 tumors than in 67NR tumors (Figure 1F). On the other hand, the level of vascular endothelial growth factor (VEGF) had a tendency to be lower in 4T1 tumors on day 33 than in 67NR tumors (Figure 1F). Plasma levels of soluble P-selectin, E-selectin, vascular cell adhesion molecule 1 (V-CAM-1), and insulin-like growth factor 1 (IGF-1) were similar or lower in mice bearing 4T1 tumors than in mice bearing 67NR tumors; however, plasma levels of soluble intercellular adhesion molecule 1 (sI-CAM-1), VEGF, and endothelin-1 (ET-1) were significantly higher in mice bearing 4T1 tumors than in mice bearing 67NR tumors (Figure 2).

Figure S1B shows changes in basic blood morphological parameters during the progression of 67NR and 4T1 tumors. The 4T1 breast cancer-bearing mice exhibit higher levels of leukocytes (including lymphocytes, monocytes and granulocytes) than 67NR-bearing mice (Figure S1B).

In summary, metastatic 4T1 tumors show that impaired blood flow and blood vessels in these tumors are more permeable. In addition, higher TGF-β1, TSP-1, VEGF, and TNF-α levels are observed in 4T1 than in 67NR tumor tissue. Plasma level of sP-selectin, sE-selectin, sV-CAM and IGF-1 is decreased, whereas the level of sI-CAM, VEGF and ET-1 is elevated in mice bearing 4T1 as compared to 67NR tumors.

### 2.2. Increased RBP4 Protein Level in Young and Aged Mice and in Patients with Breast Cancer with Metastatic and Nonmetastatic Tumors

**Figure 3:**
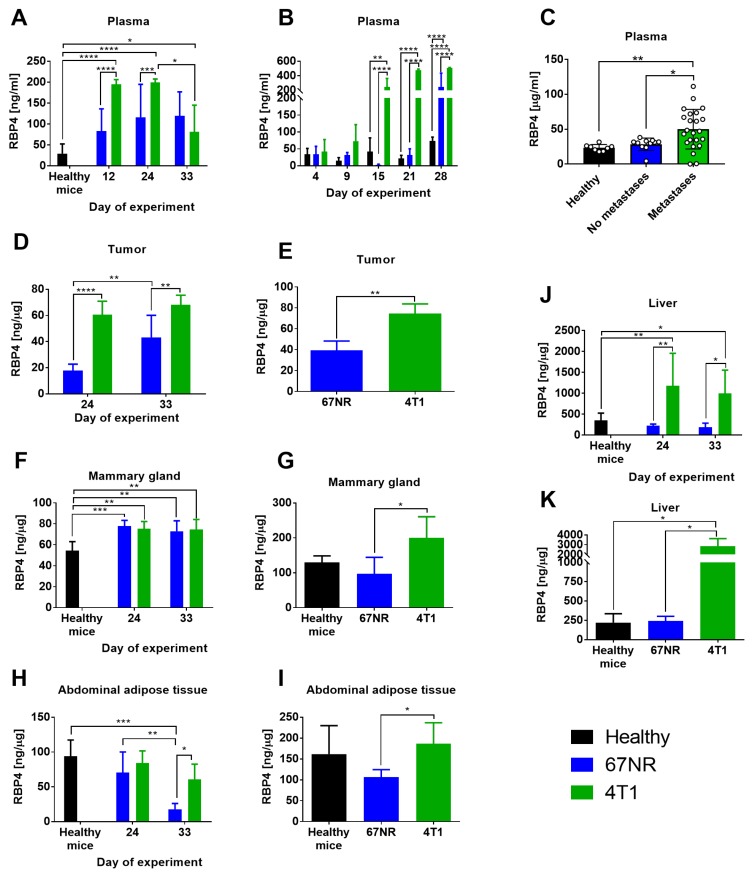
The level of RBP4 protein in plasma and various tissues from mice bearing nonmetastatic 67NR and metastatic 4T1 mammary gland cancer cells and in plasma from patients with breast cancer. Plasma from (**A**) young and (**B**) aged mice. (**C**) Plasma from patients with breast cancer. Tumors from (**D**) young and (**E**) aged mice. Mammary glands from (**F**) young and (**G**) aged mice. Abdominal adipose tissue from (**H**) young and (**I**) aged mice. Liver from (**J**) young and (**K**) aged mice. Data presented as mean ± SD or data for individual patients (Figure **C**). Number of mice per group: (**A**) 4–5; (**B**) 4 (2 healthy mice); (**D**) 5; (**E**) 4; (**F**) 5; (**G**) 4; (**H**) 4; (**I**) 3–4; (**J**) 5; (**K**) 3–4. Statistical analysis: Tukey’s multiple comparison test * *p* < 0.05, ** *p* < 0.01, *** *p* < 0.001, **** *p* < 0.0001.

Young (6–8-week-old) and aged (1-year-old) mice were ort. injected with both cell lines (4T1 and 67NR). Plasma level of RBP4 protein significantly increased in young and aged 4T1 tumor-bearing mice starting from approximately 2 weeks after cell transplantation and was significantly higher than that in 67NR tumor-bearing mice. Although the plasma level of RBP4 was also increased in 67NR tumor-bearing mice, the increase was not significant in young mice and was significant in aged mice only on the last day of observation (Figure 3A,B, respectively).

The plasma level of RBP4 in patients with breast cancer was significantly higher in specimens from metastatic cancers (lymph nodes or disseminated metastases) than those in healthy volunteers and patients without diagnosed metastases (Figure 3C). The level of RBP4 protein was elevated in the tumor tissue of young and aged 4T1 tumor-bearing mice as compared to that in 67NR tumor-bearing mice (Figure 3D,E) and in the liver tissue of 4T1 tumor-bearing mice as compared to that of control healthy mice (Figure 3J,K). In mammary gland adipose tissue from young mice, the level of RBP4 increased significantly in mice bearing both tumors as compared to that in healthy mice (Figure 3F), whereas in aged mice, the level of RBP4 increased only in mice bearing 4T1 cells (Figure 3G). In abdominal adipose tissue, we observed a higher level of RBP4 in mice bearing 4T1 cells than in mice bearing 67NR cells, but the level of this protein did not differ significantly as compared to that in healthy mice (Figure 3H,I). The exception is its significant decrease in young mice bearing 67NR tumors at the end of observation (Figure 3H). In general, the levels of RBP4 were higher in old mice than in young mice.

The levels of transthyretin (TTR; complex formation of RBP4 with TTR prevents extensive loss of RBP4 by renal filtration [31,32]) in the plasma, tumor tissue, and mammary gland (day 24) were lower in 4T1 tumor-bearing mice than in 67NR tumor-bearing mice, and the TTR levels in the liver and abdominal tissue did not differ significantly between these groups of mice (Figure S2). Figure S3 presents the results of the plasma and tumor tissue levels of RBP4 (tumor tissue), TTR, ET-1, and IGF-1 in patients and healthy volunteers. The values did not differ significantly among healthy volunteers and the analyzed groups of patients.

In summary, the plasma level of RBP4 in young and old mice bearing metastatic 4T1 cancer is higher compared to mice with non-metastatic 67NR. Similar observations are made in patients’ plasma. In addition, we observe a higher level of RBP4 in tumor, liver, and adipose tissue (young and old mice), and mammary glands (old mice) of 4T1-bearing mice when compared to 67NR.

### 2.3. Intravenous Injection of RBP4 Increases Settlement of Breast Cancer Cells in the Lungs

**Figure 4:**
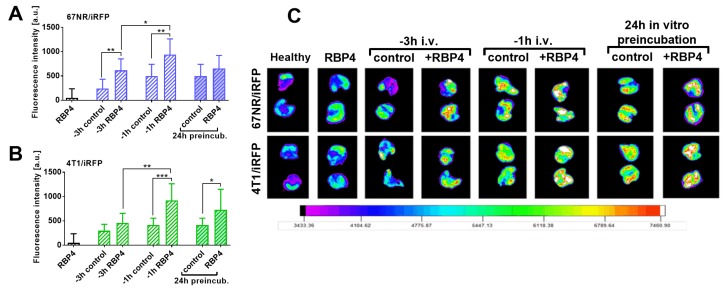
Lung fluorescence images after injection of 67NR/iRFP and 4T1/iRFP cells preceded by RBP4 protein administration. (**A**) 67NR/iRFP or (**B**) 4T1/iRFP cells were inoculated intravenously (i.v.). Three (-3 h) or one (-1 h) hour before cell inoculation, mice were administered i.v. with 500 ng/mouse of RBP4 or with saline. Alternatively, the cells were incubated with 200 ng/mL of RBP4 for 24 h and then inoculated i.v. (24 h preincubation). The lungs from healthy mice inoculated i.v. with saline were used as a reference for fluorescence measurements. Healthy mice injected with 500 ng/mouse of RBP4 were used as an additional control (RBP4). Lung fluorescence measurements were performed 48 h after cell inoculation. (**C**) Fluorescence of representative lungs from all groups is presented. No. of mice per group: healthy and RBP4 = 5, -3 h control = 6, remaining groups = 8–10. Data presented as mean ± SD. Statistical analysis: Unpaired t-test * *p* < 0.05, ** *p* < 0.01, *** *p* < 0.001.

Figure S4A shows the kinetics of RBP4 protein in mice plasma after intravenous injection of 500 ng/mouse of RBP4. The highest concentration of RBP4 was observed between 15 min and 1 h, which then gradually decreased to the basal value after 8 h. Thus, up to 1 h, the plasma level of RBP4 reached a maximum and then started to decrease. Activation of endothelium measured as an increase of P-selectin expression on mouse endothelial cells in vitro (after incubation with 200 ng/mL of RBP4) reaches a maximum after 3 h of incubation and remains at a high level for up to 5 h (Figure S4B). On the basis of these results, we planned the following experiment (Figure 4).

The 67NR/iRFP or 4T1/iRFP cells injected i.v. 1 h after the administration of 500 ng/mouse RPB4 protein were stopped significantly in the lungs (Figure 4). This influence of RBP4 protein was also observed at 3 h after its injection, but only in the case of 67NR/iRFP cells (Figure 4A). In vitro 24 h preincubation of cancer cells with RBP4 significantly increased lung settlement of 4T1 cells (Figure 4B).

### 2.4. Increase in Metastatic Potential and Tumor Blood Vessel Impairment in Mice Bearing RBP4-transduced Cells 

**Figure 5:**
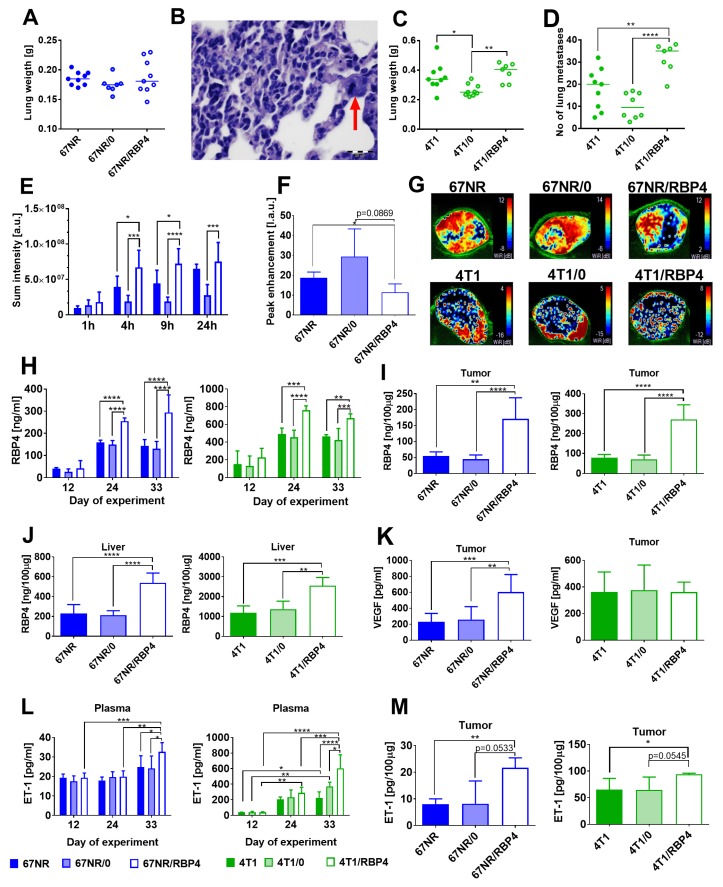
The effect of RBP4 overexpression on metastatic potential and angiogenesis of 67NR and 4T1 tumors. (**A**) Lung weight of 67NR tumor-bearing mice (*N* = 7–9) and (**B**) microphotograph of lung metastasis in mice bearing 67NR/RBP4 cells. Red arrow indicates epithelial cell with mitotic spindle. (**C**) Lung weight and (**D**) number of lung metastatic foci in mice bearing 4T1/RBP4 tumors (*N* = 7–9). (**E**) Blood vessel permeability in 67NR/RBP4 tumors (*N* = 4). (**F**) Peak enhancement in tumor tissue of mice bearing 67NR/RBP4 tumors (*N* = 3–4). (**G**) Representative pictures of wash in rate parameter. Concentration of RBP4 protein in (**H**) plasma (*N* = 3–5), (**I**) tumor tissue (*N* = 5), and (**J**) liver (*N* = 5). (**K**) VEGF in tumor tissue (*N* = 6–9). Concentration of endothelin-1 (ET-1) in (**L**) plasma (*N* = 3–4) and (**M**) tumor tissue (*N* = 3–4). Data presented as mean ± SD or data for individual measurements (Figures (**A**), (**C**), and (**D**)). Statistical analysis: Tukey’s multiple comparison test. * *p* < 0.05, ** *p* < 0.01, *** *p* < 0.001, **** *p* < 0.0001.

Both 67NR/RBP4 and 4T1/RBP4 cells with overexpression of RBP4 protein when transplanted ort. showed similar kinetics of tumor growth as compared to wild-type tumors (Figure S5A,B). Only tumors growing from 67NR/0 cells exhibited slow growth kinetics; therefore, the analyses of plasma and tissues and angiogenesis assessment were performed 10 days later, when tumor volumes of 67NR/0 tumors were comparable to those of wild-type and 67NR/RBP4 tumors (Figure S5A). Lung weight of mice bearing 67NR/RBP4 cells did not change as compared to that of controls; however, histopathological analysis revealed the presence of tumor cells in the lung tissue of 3/9 mice bearing 67NR/RBP4 cells (Figure 5A,B).

The same analysis of lungs from mice with 67NR and 67NR/0 cells did not reveal the presence of cancer cells in the lung. Lung weight and the number of metastatic foci significantly increased in mice bearing 4T1/RBP4 cells as compared to those in mice bearing 4T1/0 cells (Figure 5C,D). Moreover, we observed the impairment of tumor tissue angiogenesis (increased blood vessel permeability and decreased blood flow) when mice were transplanted with 67NR/RBP4 tumors (Figure 5E–G) or to a lesser extent even with transplantation of 4T1/RBP4 cells (Figure S6). The RBP4 protein level was significantly increased in plasma (Figure 5H), tumor tissue (Figure 5I), and liver (Figure 5J) of mice bearing 67NR/RBP4 or 4T1/RBP4 cells as compared to that in mice inoculated with cells not overexpressing RBP4. The RBP4 protein level in mammary gland and abdominal adipose tissue of mice bearing cells overexpressing RBP4 protein did not differ significantly as compared to that in control animals (Figure S5A,B). The level of VEGF in tumor tissue increased significantly, but only in mice bearing 67NR/RBP4 cells (Figure 5K). ET-1 level in plasma (Figure 5L) and tumor tissue (Figure 5M) was significantly higher in mice bearing 67NR/RBP4 or 4T1/RBP4 cells than in appropriate controls.

In summary, overexpression of RBP4 causes impaired blood flow in tumors and an increase in vascular permeability. Elevated RBP4 levels are observed in tumors, liver and plasma of mice inoculated with *Rbp4*-transduced cells. Increased ET-1 level is noticed in plasma and tumors and VEGF in tumors of mice transplanted with RBP4-overexpressing cells.

### 2.5. RBP4 Increases the Invasive Potential of 67NR and 4T1 Mouse Mammary Gland Tumor Cells In Vitro

**Figure 6:**
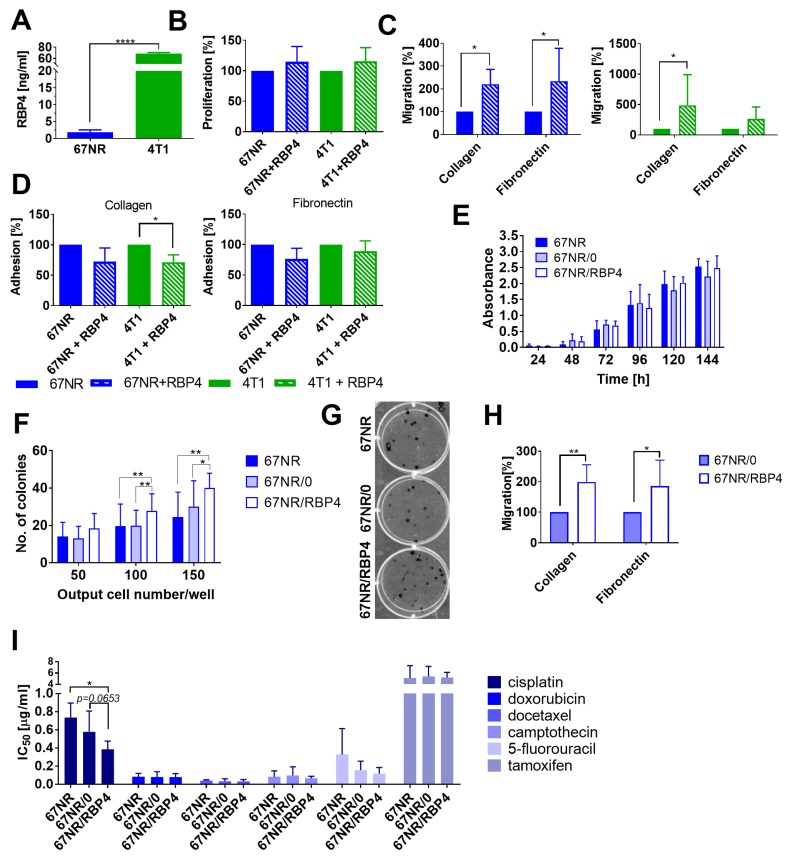
In vitro characteristics of the effect of RBP4 on 4T1 and 67NR mouse mammary gland cancer. (**A**) Comparison of RBP4 level in 4T1 and 67NR cell lysates using ELISA. (**B**–**D**) The effect of 24 h incubation with 200 ng/mL of RBP4 on (**B**) cell proliferation (*N* = 4), (**C**) migration (*N* = 6), and (**D**) adhesion (*N* = 3). (**E**) The proliferation of wild-type and transduced 67NR cells measured between 24 and 144 h (*N* = 2–6). (**F**) Number of colonies formed 14 days after seeding three different numbers of wild-type and transduced 67NR cells (*N* = 3 for 150 cells; *N* = 8 for 50 and 100 cells/well). (**G**) Representative image of colonies formed after seeding of 100 cells/well. (**H**) Migration of 67NR/0 and 67NR/RBP4 cell lines through collagen and fibronectin (*N* = 4–6). (**I**) The sensitivity of 67NR/RBP4 cells to commonly used anticancer drugs (*N* = 3–7). Statistical analysis: Tukey’s multiple comparison test or unpaired t test; (**I**) Dunnett’s multiple comparison test. * *p* < 0.05, ** *p* < 0.01, **** *p* < 0.0001.

The 4T1 cell lysates from in vitro culture showed significantly higher level of RBP4 protein than 67NR cell lysates (Figure 6A).

Incubation of both cell lines with 200 ng/mL of RBP4 did not significantly influence the proliferation rate of cells (Figure 6B). The migration of 67NR cells through collagen and fibronectin and of 4T1 cells through collagen was enhanced by RBP4 (Figure 6C). Moreover, the adhesion of 4T1 cells was inhibited significantly after incubation with RBP4 (Figure 6D). However, the expression levels of E- and N-cadherin, CD44, CD29, CD61, CD162, CD51, CD24, and CD41 did not change significantly after incubation with RBP4 (Figure S7).

Because 67NR cells seem to be more sensitive to the effect of RBP4 in vitro, we also analyzed proliferation, colony formation, and migration using the 67NR/RBP4 cell line (Figure 6E–H). Proliferation of wild-type 67NR, 67NR/0, and 67NR/RBP4 cell lines did not differ significantly (Figure 6E). However the long-term colony formation assay showed significant improvement in the number of colonies formed for the 67NR/RBP4 cell line as compared to that for both 67NR and 67NR/0 cell lines (Figure 6F,G). Migratory properties of 67NR/RBP4 cells through collagen and fibronectin significantly increased as compared to that of 67NR/0 cells (Figure 6H). Overexpression of RBP4 did not significantly influence the sensitivity of 67NR/RBP4 cells to the selected anticancer agents (Figure 6I). Except for cisplatin, we observed the tendency of increased antiproliferative activity of cisplatin against 67NR/RBP4 cells as compared to that against 67NR/0 cells (*p* = 0.0653). We also assessed the effect of RBP4 overexpression on STAT3 phosphorylation in 67NR/RBP4 cells in vitro (as well as in 67NR/RBP4 and 4T1/RBP4 tumor cell lysates), and we did not observed significant differences between wild-type or empty vector-transduced cell lines and *Rbp4*-transduced cells (Figure S8A–C). The expression of VEGF did not differ between 67NR/RBP4 cell lines and control cell lines (Figure S8D).

Exogenous RBP4 added to cell culture, as well as transfection of cells with *Rbp4*, do not affect cell proliferation but increase their migration. Overexpression of RBP4 increases the clonogenic potential of cells and may sensitize cells to cisplatin.

## 3. Discussion

Although several studies have presented the analysis of the effect of RBP4 on several types of cancers, only one study has shown such research on patients with breast cancer [25]. In their case-control study, Jiao et al. showed that serum RBP4 levels were positively associated with breast cancer risk among patients with lower BMI (<25 kg/m^2^) and that patients with ER- or PR-negative tumors possessed significantly higher serum levels of RBP4 [25]. A similar tendency (in the case of ER) was observed in our studies (Figure S3H). However, in contrast to the studies of Jiao et al., our results showed significant differences in the plasma levels of RBP4 between patients with metastatic and nonmetastatic tumors [25]. Moreover, our animal studies on two sister cell lines, nonmetastatic 67NR and metastatic 4T1, confirmed significantly higher RBP4 plasma levels in mice bearing metastatic tumors than in mice bearing nonmetastatic tumors. Moreover, the overexpression of RBP4 in cancer cells further increased the metastatic potential of the 4T1/RBP4 cell line, and for the nonmetastatic 67NR cell line [4], we could detect cancer cells in the lungs of 67NR/RBP4 tumor-bearing mice.

RBP4 protein is reported to induce endothelial inflammation through the stimulation of expression of proinflammatory molecules involved in leukocyte recruitment and adherence to the endothelium, including V-CAM-1, I-CAM-1, and E-selectin [12,13]. Endothelial inflammation and/or prolonged activation during obesity and cancer lead to endothelial dysfunction and are among the factors facilitating tumor progression and metastasis [15,33]. ET-1 is a useful and sensitive marker of endothelial dysfunction [34,35]. We observed an elevated level of ET-1 in 4T1 metastatic tumors as compared to that in 67NR tumor; moreover, the overexpression of RBP4 led to further elevation of its plasma level in mice bearing 67NR/RBP4 or 4T1/RBP4 tumors, indicating increasing endothelial dysfunction with increasing RBP4 expression in tumor cells. By using the model of bovine vascular aortic endothelial cells (BAECs), Takebayashi et al. showed that RBP4 inhibited insulin-stimulated secretion of ET-1 and induced NO production [14]. However, these interesting effects observed in their paper should be described as acute, whereas our studies showed a systematic increase in RBP4 levels in the plasma of mice, indicating chronic exposure of endothelium to its effects. Moreover, increased ET-1 levels were observed in our studies at later steps of tumor progression (24–33 days), i.e., after a significant increase of RBP4 plasma level (day 12).

The other soluble factor related to endothelial activation, namely sI-CAM-1, was elevated in plasma of mice bearing metastatic tumor as compared to that in mice bearing nonmetastatic mammary gland tumor. On the other hand, the plasma levels of sV-CAM-1, sE-selectin, and sP-selectin were decreased in metastatic tumors as compared to those in nonmetastatic tumors. The expression and shedding of all these molecules are enhanced in angiogenesis-associated diseases by angiogenic mediators released by tumor and inflammatory cells. In turn, these soluble molecules can stimulate neovascularization [36,37,38,39]. Two studies conducted in vitro showed that exogenous RBP4 can induce the expression and shedding of V-CAM, I-CAM, and E-selectin, and this activity was described to be realized through the activation of NADPH oxidase and nuclear factor κB (NF-κB) [12] or by TLR4 and in part by the JNK and p38 MAPK signaling pathways [13]. We cannot exclude the influence of various other factors/molecules whose expression was reported to differ between 4T1 and 67NR tumors on the expression of these proteins [4]. However, the final effect on angiogenesis is unambiguous, namely dysfunctional blood vessel network in highly metastatic tumors (4T1 and 4T1/RBP4) and increased blood vessel permeability with decreased blood flow in 67NR/RBP4 tumors overexpressing RBP4. The increased level of VEGF in the plasma of 4T1 tumor-bearing mice and in the tumor tissue of 67NR/RBP4 tumors also contributes to this final effect. VEGF, the main proangiogenic molecule, is responsible for excessive angiogenic response within the tumor tissue, and antiangiogenic therapies directed against this molecule or its receptors result in the normalization of the blood vessel network [40]. TGF-β, which was increased in the tumor tissue of 4T1 tumor-bearing mice, is another molecule known to affect tumor angiogenesis [41,42], and activated endothelial cells are also characterized by increased TGF-β production [43]. Therefore, the endothelium activated by RBP4 may also lead to the increased expression of this molecule. TGF-β is also the main activator of the epithelial–mesenchymal transition (EMT) process during cancer progression, in which epithelial cells break down their junctional structures, begin to express mesenchymal cell proteins, remodel their extracellular matrix, and migrate [44].

TNF-α (whose expression was higher in 4T1 tumors than in 67NR tumors) is known to be a promoter of invasion and metastasis through the activation of NF-κB signaling [45], and RBP4 can activate NF-κB [12]. Therefore, both RBP4 and NF-κB may lead to a synergistic increase in the tumor progression and metastasis process observed in our studies. The potential role of NF-κB signaling in the mechanism of the effects of RBP4 observed in our studies may be supported by the observation that NF-κB regulates the expression of VEGF (and thus tumor angiogenesis) [46]. The overexpression of RBP4 increased the level of VEGF in the tumor tissue of 67NR/RBP4, but not in the cell culture of these cells (Figure S8D). We can therefore assume that this effect is not dependent on the direct effect of RBP4 on cancer cells.

It is also known that RBP4 production is downregulated in human adipocytes by TNF-α [47]. Therefore, in our studies using wild-type tumors, the increased expression of TNF-α may be responsible for the observed lower levels of RBP4 in the plasma and abdominal adipose tissue of young mice in the last days of observation. Such effects were not observed in older mice bearing transduced cells as well as in aged mice bearing wild-type 4T1 tumors. Moreover, the plasma levels of RBP4 were higher in aged mice bearing cancer than in young mice. RBP4 expression increases during obesity, and a previous study suggested that the development of obesity leads to the increased expression of RBP4 by adipocytes [48]. Although RBP4 plasma levels did not differ significantly between healthy young 6-week-old (weighing about 20 g) and 52-week-old (about 25 g) female BALB/c mice, 4T1 tumor growth induced higher levels of RBP4 in the plasma of aged mice (about 200 ng/mL in young vs. 600 ng/mL in aged mice). Interestingly, the tumor tissue level of RBP4 did not differ between young and aged mice, but was again higher in mammary glands, abdominal adipose tissue, and liver of aged mice, similar to that observed in the plasma. Interestingly, tumors overexpressing RBP4 led to increased (as compared to that in wild-type or transduced with control vector cell lines) level of RBP4 only in the liver (besides tumor tissue and plasma) and not in the mammary gland or abdominal adipose tissue (Figure S5). Thompson et al. reported that hepatocytes are the main source of circulating RBP4 in mice and RBP4 produced by adipocytes may have a more important autocrine or paracrine function [49]. Recent studies reported that IL-6 is an important modulator of RBP4 production in the liver. Mohd et al. proposed a new mechanism involving peroxisome proliferator-activated receptor α (PPARα) and different CCAAT/enhancer binding protein (C/EBP) isoforms necessary for the regulation of *RBP4* gene expression in response to external stimuli, like IL-6, during physiological changes [50]. However, further research is required to understand the mechanisms by which growing tumors enhance RBP4 levels in other tissues and the difference observed between young and aged mice.

Apart from the influence of RBP4 in vitro on endothelial cells [12,13,14], RBP4 has also been reported to influence cancer cells [18,26]. Wang et al. showed that RBP4 can drive ovarian cancer cell migration and proliferation through the RhoA/Rock1 and ERK pathways [26]. Exogenous RBP4 and RBP4 overexpression resulted in increased migration of 4T1 or 67NR cells through collagen or fibronectin, but we did not observe any effect of RBP4 on E- and N-cadherins and other adhesion molecules analyzed (Figure S7). We also observed that 67NR/RBP4 cells possessed increased ability to form colonies from a single cell. On the other hand, an increase in MMP2 and MMP9 expression was observed in ovarian cancer cell lines in parallel with increased migratory potential of these cells [26], and downregulation of STRA6 or RBP4 in colon cancer cells decreased the fraction of cancer stem cells and tumor initiation frequency through mechanisms dependent on the activation of the STRA6 receptor by bound RBP4 and further transduction of the JAK2-STAT3 signaling cascade [18]. These mechanisms could also be important in our studies on breast cancer cells. Therefore, we analyzed the phosphorylation status of STAT3 in tumor tissue and cell culture and found that it did not change with the overexpression of RBP4 (Figure S8).

The abovementioned effects of RBP4 on metastasis and angiogenesis may therefore rely on the direct effects of RBP4 on cancer cells and endothelial cells. To show which of these effects prevail, we conducted studies by performing intravenous injection of cancer cells. Our initial research showed that endothelial cell activation in vitro (measured as expression of P-selectin) was the highest after 3 h, and at the same time, the plasma level of RBP4 injected i.v. persisted at the highest level between 15 and 60 min and then rapidly diminished (Figure S4). Therefore, we assumed that injecting RBP4 1 h prior to the injection of cancer cells may allow to observe the combined effects of RBP4 on cancer cells and endothelial cells. On the other hand, when cancer cells were injected 3 h after RBP4 injection, only the effect of RBP4 on the endothelial cells could be observed. The incubation of cancer cells for 24 h before intravenous injection should at least represent the effect of RBP4 on cancer cells. All these experimental schedules resulted in the increase of lung settlement by cancer cells, indicating that the effect of RBP4 on both cancer and endothelial cells is important. Moreover, the highest number of cancer cells in the lungs were observed in the experiment where both effects occurred: injection of cancer cells 1 h after RBP4 injection.

Increased in vitro sensitivity of 67NR/RBP4 cells to cisplatin (reduced IC_50_ value) is possibly the only beneficial property of RBP4 observed in our studies. It should be emphasized that increased levels of RBP4 in patients with diabetes are considered as a marker of renal tubular dysfunction [51]. In addition, high levels of RBP4 were also observed in patients with kidney graft dysfunction [52]. Other authors have also shown that cisplatin increases RBP4 expression in mice by inducing kidney damage [53]. Similar relationships were observed for the platinum-based drug LA-12 in both rats and patients [54]. However, our initial in vitro studies indicate that RBP4 alone does not adversely affect the sensitivity of cancer cells to anticancer drugs, including those based on platinum or other drugs that lead to kidney damage and may cause an increase in RBP4. Further research is needed to confirm these observations.

## 4. Materials and Methods 

### 4.1. Cell Lines and Cell Culture

Mouse mammary gland cancer cell line 67NR was obtained from Barbara Ann Karmanos Cancer Institute (Detroit, MI, USA) and 4T1 cell line from ATTC (Rockville, MD, USA). Variants of these cell lines transduced with near-infrared fluorescent protein iRFP670 (KC991142.1)–67NR/iRFP and 4T1/iRFP–and RBP4 protein (NM_011255.3)–67NR/RBP4 and 4T1/RBP4–as well as cells with empty vector–67NR/0 and 4T1/0–were produced using the pRRL-cppt-CMV-ires-puro-PRE-sin lentiviral vector kindly provided as part of the lentivirus system by Dr. Didier Trono (Ecole Polytechnique Fédérale de Lausanne, Lausanne, Switzerland). The efficacy of transduction was presented in Figure S4C (iRFP670) and Figure S5C (RBP4).

For lentivirus production and packaging, Lenti-X™ 293FT cells (Clontech, Mountain View, CA, USA) were cotransfected at 60% confluence with 20 μg pRRL-cppt-CMV-RBP4-ires-puro-PRE-sin, 10 μg pMDL-g/p-RRE, 5 μg pRSV-REV, and 5 μg pMk-VSVG (D. Trono, École Polytechnique Fédérale de Lausanne, Lausanne, Switzerland) using polyethylenimine (Polysciences Inc., Warrington, PA, USA) at a concentration of 1 mg/mL dissolved in phosphate-buffered saline (PBS). The virus-containing supernatant was concentrated 100× on an Amicon Ultra-15K:100.000 (Millipore, Billerica, MA, USA). The 4T1 and 67NR cells (2.5 × 10^4^) were transduced with the concentrated virus stock by centrifugation (2460× *g*) at 24 °C for 2.5 h.

67NR cells were cultured in high-glucose Dulbecco’s Modified Eagle’s Medium (DMEM; Thermo Fisher Scientific, Waltham, MA, USA) supplemented with 10% fetal bovine serum (FBS) + Fe, 2 mM L-glutamine, and 1% MEM. 4T1 cells were cultured in the 1:1 mixture of RPMI1640 + Opti-MEM medium with 5% FBS (Thermo Fisher Scientific), 2 mM L-glutamine, 4.5 g/L glucose, 1 mM sodium pyruvate (all from Sigma-Aldrich, St. Louis, MO, USA). Both culture media were supplemented with 100 µg/mL streptomycin and 100 U/mL penicillin (both from Polfa Tarchomin S.A., Warszawa, Poland). Culture media for transfected cells were supplemented with puromycin (8 µg/mL for 67NR cells and 1 µg/mL for 4T1 cells; Thermo Fisher Scientific). The Lenti-X™ 293FT cell line was maintained in high-glucose DMEM (Gibco, Scotland, UK) supplemented with 1% MEM (Sigma-Aldrich), 100 U/mL penicillin, 100 mg/mL streptomycin (both from Polfa Tarchomin S.A, Warszawa, Poland), 1 mM sodium pyruvate, 5% FBS (HyClone, Logan, UT, USA), and 6 mm L-glutamine.

### 4.2. In Vivo Experiments

The experiments were carried out on 6-week-old (about 20 g), 16-week-old (about 22 g), and 52-week-old (about 25 g) female BALB/c mice, under protocol Nos. 46/2013, 44/2016, 75/2017, and 09/2018 approved by the Local Ethical Committee for Experiments on Animals in Wroclaw, Poland. All experiments were conducted in accordance with the Directive of the European Parliament and Council No. 2010/63/EU on the protection of animals used for scientific purposes. Mice were obtained from the Animal Facility of the Experimental Medicine Center of the Medical University of Bialystok, Poland. The mice were maintained under the conditions of a 12-h day/night cycle with unrestricted access to food and drinking water.

#### 4.2.1. Cell Transplantation

The cells (67NR, 67NR/0, 67NR/RBP4: 2 × 10^6^ cells/mouse; 4T1, 4T1/0, 4T1/RBP4: 0.2 × 10^6^ cells/mouse) from in vitro culture were injected orthotopically (ort.) into the mammary fat pad. After orthotopic cell injection, the mice were observed, and their body weight and tumor growth were measured. Tumor volume [mm^3^] was calculated according to the Formula (1):

(1) $$TV=\frac{1}{2}\times a^{2}\times b$$

where *TV*—tumor volume; *a*—shorter diameter; *b*—longer diameter.

At 1–3 time points (Table 1), the mice were euthanized and blood, tumor, lungs, liver, abdominal visceral adipose tissue [55], and the tissue of the healthy mammary gland from the site opposite to the tumor location site were harvested for further analyses.

Both 67NR/iRFP and 4T1/iRFP cells were injected intravenously (i.v.) into the lateral tail vein in the number of 0.6 × 10^6^ cells/mouse and mice were euthanized 48 h after cell transplantation. One or three hours before intravenous cell transplantation, the mice were injected i.v. with 500 ng/mouse of RBP4 (RBP4 Recombinant Mouse Protein, His Tag; Thermo Fisher Scientific) or with 67NR/iRFP or 4T1/iRFP cells preincubated for 24 h before transplantation with 200 ng/mL of RBP4. The details of experiments are summarized in Table 1. As a control, healthy BALB/c mice of the corresponding age were used in selected analyses.

#### 4.2.2. Tumor Angiogenesis Assessment

To compare tumor angiogenesis between 67NR and 4T1 cell lines and in mice bearing cells overexpressing RBP4 on day 26 (or in the case of slowly growing 67NR/0 cells, when tumors reached volume of 1000 mm^3^), two methods were used.

Tumor blood perfusion analysis was performed using intravenous injection of MicroMarker^TM^ Contrast Agent by the Vevo2100 ultrasound imaging system (VisualSonics, Ontario, Canada) as described previously [10]. The analysis of the received data was carried out using the VevoLab and VevoCQ software (VisualSonics). Tumor perfusion was assessed on the basis of quantitative contrast analysis in the central part of the tumor at the pixel level by calculating the perfusion parameters related to the amplitude and time according to the fit of the curve algorithm.

To evaluate vascular permeability, mice were administered i.v. (1 nmol/mouse) the IRDye^®^ 800CW fluorescent dye PEG Contrast Agent (LI-COR, Lincoln, NE, USA). IRDye^®^ 800CW selectively accumulates within the tumor tissue through increased vascular permeability and impaired lymphatic drainage in the tumor. At 1, 4, 9 and 24 h after administration, fluorescence measurements were performed. For this purpose, the animals were anesthetized by infusing a continuous 3% isoflurane mixture in synthetic air. The animals were then placed in a chamber for visualization for small rodents of the In Vivo MS FX Pro system (Carestream Health Inc., Rochester, NY, USA), equipped with individual masks for providing an anesthetic. During fluorescence imaging, the following camera settings were used: t = 30 s, f-stop = 2.8, FOV = 200 mm, excitation wavelength: 760 nm, emission wavelength: 830 nm. In addition, X-ray pictures of the examined animals were taken to allow localization of tumor. The imaging was performed using the following camera settings: t = 2 min, f-stop = 5.57, FOV = 200. The obtained fluorescence images were analyzed using the Carestream MI SE software (Carestream Health Inc.) based on analyzed regions.

#### 4.2.3. Lung Fluorescence Measurement

Measurement of fluorescence of lungs dissected during autopsy was performed using the In Vivo MS FX Pro system with coregistration of fluorescence and X-ray. The obtained fluorescence images were analyzed using the Carestream MI SE software based on analyzed regions as described previously [56].

#### 4.2.4. Blood Morphological Analyses

Whole blood was collected in a tube containing low-molecular-weight heparin (LMWH) at 5000 IU/mL and then analyzed using the hematology analyzer Mythic 18 (C2 Diagnostics, Montpellier, France).

### 4.3. Plasma and Tumor Tissue from Patients with Breast Cancer

Approval was obtained from the Bioethical Commission at the Medical University in Wroclaw for studies on plasma and tumor tissue from patients with breast cancer and healthy donors (Approval No. 71/2017). Informed consent was obtained from persons participating in the study. All procedures were conducted in accordance with the institutional and international ethical standards. Blood samples and tumor samples were collected from July to September 2017 in Wroclaw Comprehensive Cancer Center, Poland, from 34 patients at various stages of breast cancer. In addition, blood from eight healthy donors was used as a control. Blood was collected from peripheral veins into heparin-containing tubes. Patients were divided into two groups according to the stage of breast cancer: nonmetastatic and patients with metastases (in the lymph nodes and patients with distant metastases) (Table 2).

### 4.4. In Vitro Experiments

#### 4.4.1. Cell Preparation to Evaluate Proliferation, Migration, Adhesion, and Integrin Expression after Incubation with RBP4

After 24 h culture of 67NR and 4T1 cells, the culture medium was changed to medium with 5% FBS. After further 24 h, the medium was removed and replaced with a fresh medium containing 5% FBS and RBP4 protein at a concentration of 200 ng/mL. After 24 h of incubation, the cell proliferation was assessed, or the cells were harvested using nonenzymatic Cell Dissociation Solution (Sigma-Aldrich). It was then neutralized by the addition of medium, and the density of the cells was counted. Cells prepared in this way were used for further tests:

- Proliferation

The sulforhodamine B (SRB) assay was performed as described previously [57] and the percentage of cell proliferation was calculated as follows Formula (2):

(2) $$\%\;of\;proliferation=\left[100\times \left(1-\frac{Ab-Am}{Ak-Am}\right)\right]$$

where:

Ab—absorbance value measured for cells treated with RBP4
Ak—absorbance value measured for untreated cells
Am—absorbance value measured for the culture medium

- Migration

Inserts (Transwell Permeable Supports 6.5 mm Insert, Corning Incorporated, New York, NY, USA) were coated with type IV collagen or fibronectin (both from Sigma-Aldrich) at a concentration of 10 μg/mL diluted in 2% acetic acid or water, respectively, and incubated overnight at 4 °C. Subsequently, the inserts were rinsed twice with PBS and blocked with 1% BSA (Bio-Rad, Laboratories, Hercules, CA, USA) for 1 h at 37 °C. After incubation, the inserts were rinsed again with PBS and 25,000 cells suspended in 250 μL DMEM were added. The inserts were placed in the wells with culture medium and left in an incubator at 37 °C for 8 h for 4T1 and 67NR cells to migrate on collagen and for 6 h for 67NR cells to migrate on fibronectin. After incubation, the inserts were rinsed twice with PBS to remove cells that were not migrated, and the cells were stained with 0.2% crystal violet in 20% MetOH and counted using an Olympus CX microscope (Olympus Europe Holding GmbH, Hamburg, Germany).

- Adhesion

Wells of 96-well plates were coated with 10 μg/mL of collagen or fibrinogen diluted in 2% acetic acid or water, respectively, and incubated overnight at 4 °C. The next day, the plates were washed with TSM buffer (2 × 300 μL), and 100 μL of 1% BSA/TSM solution was added to block nonspecific binding sites. The plates were incubated for 30 min at 37 °C. After incubation, the plates were washed again with TSM buffer (2 × 300 μL). The cells were then plated at a concentration of 5 × 10^5^/mL in 50 μL of 0.5% BSA/TSM. The plates were incubated for 60 min at 37 °C. After incubation, the plates were washed three times with 300 μL of TSM buffer to remove nonadherent cells. Adherent cells were stained with a solution of 0.2% crystal violet in 20% MetOH in a volume of 50 μL per well. The plates were incubated for 30 min at 4 °C and then washed, and the cell suspension was diluted with 100 μL of 80% MetOH. The optical density of the samples was read using a Biotek Hybrid H4 reader (BioTek Instruments, Winooski, VT, USA) at 570 nm.

- Flow cytometry analysis

The cell pellet was suspended in PBS solution with the addition of 2% FBS. Cells were counted and 2.5–5 × 10^5^ cells were stained with antibodies for 30 min at 4 °C in the dark, centrifuged, and suspended in PBS. The analysis was performed in a BD Fortessa cytometer using the Diva software (Becton Dickinson, East Rutherford, NJ, USA).

List of antibodies used for flow cytometry analysis: BV421 Rat Anti-Mouse CD162, BV421 Rat Anti-Mouse CD41, FITC Rat Anti-Mouse CD29, FITC Rat Anti-Mouse CD44, FITC Rat Anti-Mouse CD61, PE Rat Anti-Mouse CD24, PE Rat Anti-Mouse CD51, and PE P-selectin 62P (all from BD Biosciences, San Jose, CA, USA).

#### 4.4.2. Proliferation and Migration Evaluation and Clonogenic Assay Using 67NR/RBP4 Cell Line

- Proliferation

The MTT assay was performed as described previously [57] with minor modifications. Briefly, cells were seeded at a density of 1500 cells per well in 96-well cell culture plates and maintained at 37 °C in 5% CO_2_. After incubation (24, 48, 72, 96, 120, or 144 h), 20 µL of a 5 mg/mL solution of 3-(4,5-dimethylthiazol-2-yl)-2,5-diphenyltetrazolium bromide (Sigma-Aldrich, St. Louis, MO, USA) in PBS was added to each well. The cells were then incubated at 37°C for 4 h. Then, the medium was removed, and the cells were lysed by adding 200 µL/well of DMSO (Avantor Performance Materials, Gliwice, Poland) The resulting formazan crystals were dissolved in DMSO, and absorbance at 570 nm was measured using a Biotek Hybrid H4 reader (BioTek Instruments, Winooski, VT, USA).

- Proliferation of cells treated with anticancer agents

Cisplatin, doxorubicin, docetaxel, and 5-fluorouracil were purchased from Accord Healthcare Poland (Warsaw, Poland). Camptothecin and tamoxifen were purchased from Sigma-Aldrich (St. Louis, MO, USA). The effects of anticancer drugs on the cell growth of 67NR, 67NR/0, and 67NR/RBP4 cell lines were measured using the MTT assay as described above. Briefly, cells were seeded at a density of 1500 cells per well in 96-well cell culture plates 1 day prior to the assay and maintained at 37 °C in 5% CO_2_. The cells were then treated with cisplatin, doxorubicin, 5-FU, or camptothecin at four concentrations in the range of 0.001 to 1 µg/mL, docetaxel at four concentrations in the range of 0.0001 to 0.1 µg/mL, and tamoxifen at four concentrations in the range of 0.01 to 10 µg/mL for 72 h. The solvent for camptothecin and tamoxifen (DMSO) used at the highest concentration (0.1%) in the assay did not cause any cytotoxicity. All compounds were diluted prior to use in culture medium to the required concentrations. The IC_50_ value was defined as the concentration required for half-maximal (50%) inhibition of cell growth as compared to the growth of untreated cells. The IC_50_ values were calculated based on Cheburator 0.4 software [58]. In each experiment, samples containing specific concentrations of the preparation were used in triplicate. The experiments were repeated 3–7 times.

- Migration

The inserts (prepared as described above) were placed in the wells with culture medium and left in an incubator at 37 °C for 6 h. After incubation, the cells were stained with RAL Diff-Quik kit (RAL Diagnostics, Martillac, France), rinsed twice with PBS to remove cells that had not migrated, and counted using an Olympus CX microscope (Olympus Europe Holding GmbH, Hamburg, Germany).

- Clonogenic assay

The viable cells were counted and seeded at a density of 50, 100, or 150 cells on the wells of a 6-well plate. After 7 days, the colonies were fixed and stained with 1% crystal violet/methanol (Sigma-Aldrich), documented with a ChemiDoc Imaging System (Bio-Rad Laboratories), and counted manually.

### 4.5. Tissue and Cell Lysate Preparation for ELISA and Western Blot

Frozen tissue was homogenized with an appropriate amount of RIPA buffer with a cocktail of phosphatase and protease inhibitors (all from Sigma-Aldrich, St. Louis, MO, USA) using Fast Prep^®^-24 MP Bio homogenizer (MP Biomedicals LLC, Santa Ana, CA, USA). The samples were then incubated on ice for 20 min and centrifuged (4 °C, 15 min, 12,000× *g*). The obtained supernatant was transferred to 1.5 mL tubes and again centrifuged. 

Cells plated on culture dishes were rinsed twice with PBS, and 90 μL of RIPA buffer containing a cocktail of protease and phosphatase inhibitors was added to the cells. The cells were then harvested with scrapers, transferred to tubes, and incubated on ice for 20 min. After incubation, the tubes were centrifuged for 15 min at 4 °C at 10,000× *g*.

The obtained supernatants were transferred to new tubes and stored at −80°C for further analysis. Protein content were analyzed using the Bio-Rad Protein Assay kit (Bio-Rad Laboratories).

#### 4.5.1. ELISA Tests

ELISA tests were performed according to the manufacturer’s protocols. The result of the analysis was read using a Biotek Synergy H4 Hybrid reader (BioTek Instruments, Winooski, VT, USA) by measuring the absorbance at 450 nm. Standard curves were prepared, which were used to determine the concentration of test samples.

List of ELISA kits used (anti-mouse): ET-1 (Endothelin 1), I-CAM-1/CD54 (Intercellular Adhesion Molecule 1), IGF-1 (Insulin-like Growth Factor 1), RBP4 (Retinol Binding Protein 4, Plasma), SeLE (E-selectin), SeLP (P-Selectin), sV-CAM-1/CD106 (soluble Vascular Cell Adhesion Molecule 1), TNF-α (Tumor Necrosis Factor-Alpha), TSP-1 (Thrombospondin-1), TTR (Transthyretin), TGF-β1 (TGF-beta1 (Transforming Growth Factor-beta1) (all from Elabscience Biotechnology Co, Wuhan, China); VEGF-A (Vascular Endothelial Cell Growth Factor A) (from Thermo Fisher Scientific, Waltham, MA, USA or R&D Systems, MN, USA); InstantOne ELISA STAT3 (Total/Phospho), Invitrogen (Waltham, MA, USA).

#### 4.5.2. Western Blot

List of anti-mouse protein antibodies used: anti-E-cadherin, anti-N-cadherin (both from Proteintech, Chicago, IL, USA), anti-RBP4 (Abcam, Cambridge, UK), and anti-β-actin-horse radish peroxidase (HRP) (Santa Cruz Biotechnology, Inc., Heidelberg, Germany). Equal amounts of protein (50 μg of cell culture lysates) were mixed with 4× Laemmli Sample Buffer (Bio-Rad Laboratories, Hercules, CA, USA). Then, the samples were separated in a 4–20% sodium dodecyl sulfate (SDS)-polyacrylamide gel and transferred to a polyvinylidene difluoride (PVDF) membrane (0.45 µm; Merck Millipore, city, state abbrev, USA). The membranes were blocked for 1 h at room temperature in 5% non-fat dry milk in 0.1% PBS/Tween-20 (PBST). Next, the membranes were washed (3 × 10 min) with 0.1% PBST and then incubated overnight at 4 °C with a primary antibody. After incubation, the membranes were washed (3 × 10 min) with 0.1% PBST and incubated for 1 h with the secondary mouse anti-rabbit immunoglobulin G (IgG)-HRP antibody (Santa Cruz Biotechnology Inc., Santa Cruz, CA, USA). The membranes after washing with 0.1% PBST were detected by the ECL method. Chemiluminescence was visualized using Image Station 4000MM PRO (Carestream Health Inc., Rochester, NY, USA). Densitometry analysis of the blots was performed using Carestream MI Software 5.0.6.20 (Carestream Health Inc., Rochester, NY, USA).

### 4.6. Statistical Analysis

Statistical analysis of the results was performed using GraphPad Prism 7. The data normality analysis was performed using the Shapiro-Wilk data normality test assuming the significance of the test for *p* < 0.05. Statistical analysis for normal distribution data was performed using the ANOVA test. When the ANOVA test showed significant differences between the groups under consideration, further analyses were performed using Tukey’s test or Sidak’s test for multiple comparisons. In the event that the data distribution differed from normal, the analysis was conducted using the Kruskal-Wallis test for multiple comparisons. In some cases, the Mann–Whitney test or the t test was applied depending on the data distribution. Differences between the groups were considered statistically significant at *p* < 0.05.

## 5. Conclusions

The RBP4 protein may be an important driver of metastasis and angiogenesis of breast tumors. It affects endothelial cells by increasing the symptoms of endothelial dysfunction/activation and dysfunctional tumor angiogenesis. Furthermore, the direct effect of RBP4 on cancer cells through increased migratory and colony-forming properties contributes to the final prometastatic effect. The effect of RBP4 on tumor tissue and cancer cells in this model is not dependent on STAT3 phosphorylation.

## Figures and Tables

**Table 1:** Detailed characteristics of animal experiments.

Cell Line	Route of Transplantation	Age of Mice (weeks)	Time Points of Euthanasia—Days after Cell Transplantation
67NR, 4T1	ort.	6	12, 24, 33, “*26*”
67NR, 4T1	ort.	52	28 (4, 9, 15, 21) *
67NR, 67NR/0, 67NR/RBP4, 4T1, 4T1/0, 4T1/RBP4	ort.	16	12, 24, 33 **, “*26*”
67NR/iRFP and 4T1/iRFP	i.v.	16	48 h after cell transplantation

* in brackets: days of blood collection from zygomatic vein; ** 67NR/0 cell line: last time point of euthanasia 43 day; “*26*”—day of angiogenesis assessment; except for 67NR/0: the day when tumor reached the volume of 1000 mm^3^, comparable to 67NR and 67NR/RBP4 on day 26; ort.—orthotopically; i.v.—intravenously.

**Table 2:** Characteristics of patients with breast cancer.

Patients	No.	Age: Median (min-max)	Diabetes	Tumor Diameter: Mean ± SD (mm)	Ki67: Median (min-max) [%]	ER+	PR+	HER2+
No metastases	10	62 (47–85)	0/10	25 ± 23	20 (1–50)	9/10	7/10	8/10
Metastases	24	57 (31–83)	5/24	32 ± 21	15 (3–60)	14/24	13/24	13/24

## Supplementary Materials

The following are available online at https://www.mdpi.com/2072-6694/12/3/623/s1, Figure S1. Selected blood morphological parameters of mice bearing 67NR and 4T1 tumors. Figure S2. The level of TTR protein in plasma and various tissues from mice bearing nonmetastatic 67NR and metastatic 4T1 mammary gland cancer cells (young mice). Figure S3. Patients plasma and tumor tissue levels. Figure S4. Kinetics of plasma level of RBP4 and in vitro kinetics of endothelial cells activation after incubation with RBP4. Figure S5. The effect of RBP4 overexpression on kinetics of 67NR/RBP4 and 4T1/RBP4 tumor growth as well as the level of RBP4 in mammary gland and abdominal adipose tissue. Figure S6. The effect of RBP4 overexpression on angiogenesis of 67NR and 4T1 tumors. Figure S7. Expression of surface molecules after 24 h in vitro incubation of 67NR and 4T1 cells with RBP4 protein at a concentration of 200 ng/mL. Figure S8. STAT3 phosphorylation and VEGF expression in cell lines with overexpression of RBP4. STAT3 phosphorylation status in tumor lysates.

## References

[1] DuPré S.A., Redelman D., Hunter K.W. (2007). The mouse mammary carcinoma 4T1: Characterization of the cellular landscape of primary tumours and metastatic tumour foci. Int. J. Exp. Pathol..

[2] Aslakson C.J., Miller F.R. (1992). Selective events in the metastatic process defined by analysis of the sequential dissemination of subpopulations of a mouse mammary tumor. Cancer Res..

[3] Heppner G.H., Miller F.R., Shekhar P.M. (2000). Nontransgenic models of breast cancer. Breast Cancer Res..

[4] Johnstone C.N., Smith Y.E., Cao Y., Burrows A.D., Cross R.S.N., Ling X., Redvers R.P., Doherty J.P., Eckhardt B.L., Natoli A.L. (2015). Functional and molecular characterisation of EO771.LMB tumours, a new C57BL/6-mouse-derived model of spontaneously metastatic mammary cancer. Dis. Model. Mech..

[5] Buczek E., Denslow A., Mateuszuk L., Proniewski B., Wojcik T., Sitek B., Fedorowicz A., Jasztal A., Kus E., Chmura- Skirlinska A. (2018). Alterations in NO- and PGI2- dependent function in aorta in the orthotopic murine model of metastatic 4T1 breast cancer: Relationship with pulmonary endothelial dysfunction and systemic inflammation. BMC Cancer.

[6] Pacia M.Z., Mateuszuk L., Buczek E., Chlopicki S., Blazejczyk A., Wietrzyk J., Baranska M., Kaczor A. (2016). Rapid biochemical profiling of endothelial dysfunction in diabetes, hypertension and cancer metastasis by hierarchical cluster analysis of Raman spectra. J. Raman Spectrosc..

[7] Smeda M., Kieronska A., Adamski M.G., Proniewski B., Sternak M., Mohaissen T., Przyborowski K., Derszniak K., Kaczor D., Stojak M. (2018). Nitric oxide deficiency and endothelial–Mesenchymal transition of pulmonary endothelium in the progression of 4T1 metastatic breast cancer in mice. Breast Cancer Res..

[8] Porshneva K., Papiernik D., Psurski M., Nowak M., Matkowski R., Ekiert M., Milczarek M., Banach J., Jarosz J., Wietrzyk J. (2018). Combination Therapy with DETA/NO and Clopidogrel Inhibits Metastasis in Murine Mammary Gland Cancer Models via Improved Vasoprotection. Mol. Pharm..

[9] Blazejczyk A., Switalska M., Chlopicki S., Marcinek A., Gebicki J., Nowak M., Nasulewicz-Goldeman A., Wietrzyk J. (2016). 1-methylnicotinamide and its structural analog 1,4-dimethylpyridine for the prevention of cancer metastasis. J. Exp. Clin. Cancer Res..

[10] Porshneva K., Papiernik D., Psurski M., Łupicka-Słowik A., Matkowski R., Ekiert M., Nowak M., Jarosz J., Banach J., Milczarek M. (2019). Temporal inhibition of mouse mammary gland cancer metastasis by CORM-A1 and DETA/NO combination therapy. Theranostics.

[11] Park S.E., Kim D.H., Lee J.H., Park J.S., Kang E.S., Ahn C.W., Lee H.C., Cha B.S. (2009). Retinol-binding protein-4 is associated with endothelial dysfunction in adults with newly diagnosed type 2 diabetes mellitus. Atherosclerosis.

[12] Farjo K.M., Farjo R.A., Halsey S., Moiseyev G., Ma J.X. (2012). Retinol-Binding Protein 4 Induces Inflammation in Human Endothelial Cells by an NADPH Oxidase- and Nuclear Factor Kappa B-Dependent and Retinol-Independent Mechanism. Mol. Cell. Biol..

[13] Du M., Martin A., Hays F., Johnson J., Farjo R.A., Farjo K.M. (2017). Serum retinol-binding protein-induced endothelial inflammation is mediated through the activation of toll-like receptor 4. Mol. Vis..

[14] Takebayashi K., Sohma R., Aso Y., Inukai T. (2011). Effects of retinol binding protein-4 on vascular endothelial cells. Biochem. Biophys. Res. Commun..

[15] Jung U., Choi M.-S., Jung U.J., Choi M.-S. (2014). Obesity and Its Metabolic Complications: The Role of Adipokines and the Relationship between Obesity, Inflammation, Insulin Resistance, Dyslipidemia and Nonalcoholic Fatty Liver Disease. Int. J. Mol. Sci..

[16] Noy N., Li L., Abola M.V., Berger N.A. (2015). Is retinol binding protein 4 a link between adiposity and cancer?. Horm. Mol. Biol. Clin. Investig..

[17] Fei W., Chen L., Chen J., Shi Q., Zhang L., Liu S., Li L., Zheng L., Hu X. (2017). RBP4 and THBS2 are serum biomarkers for diagnosis of colorectal cancer. Oncotarget.

[18] Karunanithi S., Levi L., DeVecchio J., Karagkounis G., Reizes O., Lathia J.D., Kalady M.F., Noy N. (2017). RBP4-STRA6 Pathway Drives Cancer Stem Cell Maintenance and Mediates High-Fat Diet-Induced Colon Carcinogenesis. Stem Cell Rep..

[19] Abola M.V., Thompson C.L., Chen Z., Chak A., Berger N.A., Kirwan J.P., Li L. (2015). Serum levels of retinol-binding protein 4 and risk of colon adenoma. Endocr. Relat. Cancer.

[20] Cheng Y., Liu C., Zhang N., Wang S., Zhang Z. (2014). Proteomics Analysis for Finding Serum Markers of Ovarian Cancer. Biomed. Res. Int..

[21] Sobotka R., Čapoun O., Kalousová M., Hanuš T., Zima T., Koštířová M., Soukup V. (2017). Prognostic Importance of Vitamins A, E and Retinol-binding Protein 4 in Renal Cell Carcinoma Patients. Anticancer Res..

[22] El-Mesallamy H.O., Hamdy N.M., Zaghloul A.S., Sallam A.M. (2012). Serum retinol binding protein-4 and neutrophil gelatinase-associated lipocalin are interrelated in pancreatic cancer patients. Scand. J. Clin. Lab. Invest..

[23] Wang D.-D.D., Zhao Y.-M.M., Wang L., Ren G., Wang F., Xia Z.-G.G., Wang X.-L.L., Zhang T., Pan Q., Dai Z. (2011). Preoperative serum retinol-binding protein 4 is associated with the prognosis of patients with hepatocellular carcinoma after curative resection. J. Cancer Res. Clin. Oncol..

[24] Chen Y., Azman S.N., Kerishnan J.P., Zain R.B., Chen Y.N., Wong Y.L., Gopinath S.C.B. (2014). Identification of host-immune response protein candidates in the sera of human oral squamous cell carcinoma patients. PLoS ONE.

[25] Jiao C., Cui L., Ma A., Li N., Si H. (2016). Elevated serum levels of retinol-binding protein 4 are associated with breast cancer risk: A Case-Control study. PLoS ONE.

[26] Wang Y., Wang Y., Zhang Z. (2018). Adipokine RBP4 drives ovarian cancer cell migration. J. Ovarian Res..

[27] Piano A., Titorenko V.I. (2015). The Intricate Interplay between Mechanisms Underlying Aging and Cancer. Aging Dis..

[28] Meehan B., Dombrovsky A., Lau K., Lai T., Magnus N., Montermini L., Rak J. (2013). Impact of host ageing on the metastatic phenotype. Mech. Ageing Dev..

[29] Meehan B., Garnier D., Dombrovsky A., Lau K., D’Asti E., Magnus N., Rak J. (2014). Ageing-related responses to antiangiogenic effects of sunitinib in atherosclerosis-prone mice. Mech. Ageing Dev..

[30] Klement H., St Croix B., Milsom C., May L., Guo Q., Yu J.L., Klement P., Rak J. (2007). Atherosclerosis and vascular aging as modifiers of tumor progression, angiogenesis, and responsiveness to therapy. Am. J. Pathol..

[31] Zanotti G., Berni R. (2004). Plasma Retinol-Binding Protein: Structure and Interactions with Retinol, Retinoids, and Transthyretin. Vitam. Horm..

[32] Naylor H.M., Newcomer M.E. (1999). The structure of human retinol-binding protein (RBP) with its carrier protein transthyretin reveals an interaction with the carboxy terminus of RBP. Biochemistry.

[33] Blazejczyk A., Papiernik D., Porshneva K., Sadowska J., Wietrzyk J. (2015). Endothelium and cancer metastasis: Perspectives for antimetastatic therapy. Pharmacol. Reports.

[34] Endemann D.H., Schiffrin E.L. (2004). Endothelial Dysfunction. J. Am. Soc. Nephrol..

[35] Iglarz M., Clozel M. (2007). Mechanisms of ET-1-induced endothelial dysfunction. J. Cardiovasc. Pharmacol..

[36] Giavazzi R., Chirivi R.G., Garofalo A., Rambaldi A., Hemingway I., Pigott R., Gearing A.J. (1992). Soluble intercellular adhesion molecule 1 is released by human melanoma cells and is associated with tumor growth in nude mice. Cancer Res..

[37] Gho Y.S., Kleinman H.K., Sosne G. (1999). Angiogenic activity of human soluble intercellular adhesion molecule-1. Cancer Res..

[38] Morbidelli L., Brogelli L., Granger H.J., Ziche M. (1997). Endothelial cell migration is induced by soluble P-selectin. Life Sci..

[39] Koch A.E., Halloran M.M., Haskell C.J., Shah M.R., Polverini P.J. (1995). Angiogenesis mediated by soluble forms of E-selectin and vascular cell adhesion molecule-1. Nature.

[40] Maj E., Papiernik D., Wietrzyk J. (2016). Antiangiogenic cancer treatment: The great discovery and greater complexity (Review). Int. J. Oncol..

[41] Ferrari G., Cook B.D., Terushkin V., Pintucci G., Mignatti P. (2009). Transforming growth factor-beta 1 (TGF-beta1) induces angiogenesis through vascular endothelial growth factor (VEGF)-mediated apoptosis. J. Cell. Physiol..

[42] Viñals F., Pouysségur J. (2001). Transforming growth factor beta1 (TGF-beta1) promotes endothelial cell survival during in vitro angiogenesis via an autocrine mechanism implicating TGF-alpha signaling. Mol. Cell. Biol..

[43] Pintavorn P., Ballermann B.J. (1997). TGF-β and the endothelium during immune injury. Kidney Int..

[44] Moustakas A., Heldin C.-H. (2007). Signaling networks guiding epithelial-mesenchymal transitions during embryogenesis and cancer progression. Cancer Sci..

[45] Tang D., Tao D., Fang Y., Deng C., Xu Q., Zhou J. (2017). TNF-Alpha Promotes Invasion and Metastasis via NF-Kappa B Pathway in Oral Squamous Cell Carcinoma. Med. Sci. Monit. Basic Res..

[46] Xie T.-X.X., Xia Z., Zhang N., Gong W., Huang S. (2010). Constitutive NF-κB activity regulates the expression of VEGF and IL-8 and tumor angiogenesis of human glioblastoma. Oncol. Rep..

[47] Sell H., Eckel J. (2007). Regulation of retinol binding protein 4 production in primary human adipocytes by adiponectin, troglitazone and TNF-α [2]. Diabetologia.

[48] Yang Q., Graham T.E., Mody N., Preitner F., Peroni O.D., Zabolotny J.M., Kotani K., Quadro L., Kahn B.B. (2005). Serum retinol binding protein 4 contributes to insulin resistance in obesity and type 2 diabetes. Nature.

[49] Thompson S.J., Sargsyan A., Lee S.A., Yuen J.J., Cai J., Smalling R., Ghyselinck N., Mark M., Blaner W.S., Graham T.E. (2017). Hepatocytes are the principal source of circulating RBP4 in mice. Diabetes.

[50] Mohd M.A., Ahmad Norudin N.A., Muhammad T.S.T. (2020). Transcriptional regulation of retinol binding protein 4 by Interleukin-6 via peroxisome proliferator-activated receptor α and CCAAT/Enhancer binding proteins. Mol. Cell. Endocrinol..

[51] Shimizu H., Negishi M., Shimomura Y., Mori M. (1992). Changes in urinary retinol binding protein excretion and other indices of renal tubular damage in patients with non-insulin dependent diabetes. Diabetes Res. Clin. Pract..

[52] Hosaka B., Park S.I., Felipe C.R., Garcia R.G., Machado P.G.P., Pereira A.B., Tedesco-Silva H., Medina-Pestana J.O. (2003). Predictive value of urinary retinol binding protein for graft dysfunction after kidney transplantation. Transplant. Proc..

[53] Hung Y.C., Huang G.S., Lin L.W., Hong M.Y., Se P.S. (2007). Thea sinensis melanin prevents cisplatin-induced nephrotoxicity in mice. Food Chem. Toxicol..

[54] Bouchal P., Jarkovsky J., Hrazdilova K., Dvorakova M., Struharova I., Hernychova L., Damborsky J., Sova P., Vojtesek B. (2011). The new platinum-based anticancer agent LA-12 induces retinol binding protein 4 in vivo. Proteome Sci..

[55] Kong S., Ruan J., Zhang K., Hu B., Cheng Y., Zhang Y., Yang S., Li K. (2018). Kill two birds with one stone: Making multi-transgenic pre-diabetes mouse models through insulin resistance and pancreatic apoptosis pathogenesis. PeerJ.

[56] Denslow A., Świtalska M., Jarosz J., Papiernik D., Porshneva K., Nowak M., Wietrzyk J. (2017). Clopidogrel in a combined therapy with anticancer drugs—Effect on tumor growth, metastasis, and treatment toxicity: Studies in animal models. PLoS ONE.

[57] Wietrzyk J., Chodyński M., Fitak H., Wojdat E., Kutner A., Opolski A. (2007). Antitumor properties of diastereomeric and geometric analogs of vitamin D3. Anticancer. Drugs.

[58] Nevozhay D. (2014). Cheburator software for automatically calculating drug inhibitory concentrations from in vitroscreening assays. PLoS ONE.

