# Supplementary material for: Retinol-Binding Protein 4 Accelerates Metastatic Spread and Increases Impairment of Blood Flow in Mouse Mammary Gland Tumors
Source: Cancers (Basel). 2020 Mar 7;12(3):623. doi: 10.3390/cancers12030623 (PMC7139568; doi:10.3390/cancers12030623)
Supplement: Supplementary file 1 [file cancers-12-00623-s001.pdf]

Supplementary Material: Retinol-Binding Protein 4 Accelerates Metastatic Spread and Increases Impairment of Blood Flow in Mouse Mammary Gland Tumors

Diana Papiernik, Anna Urbaniak, Dagmara Kłopotowska, Anna Nasulewicz-Goldeman, Marcin Ekiert, Marcin Nowak, Joanna Jarosz, Monika Cuprych, Aleksandra Strzykalska, Maciej Ugorski, Rafał Matkowski and Joanna Wietrzyk

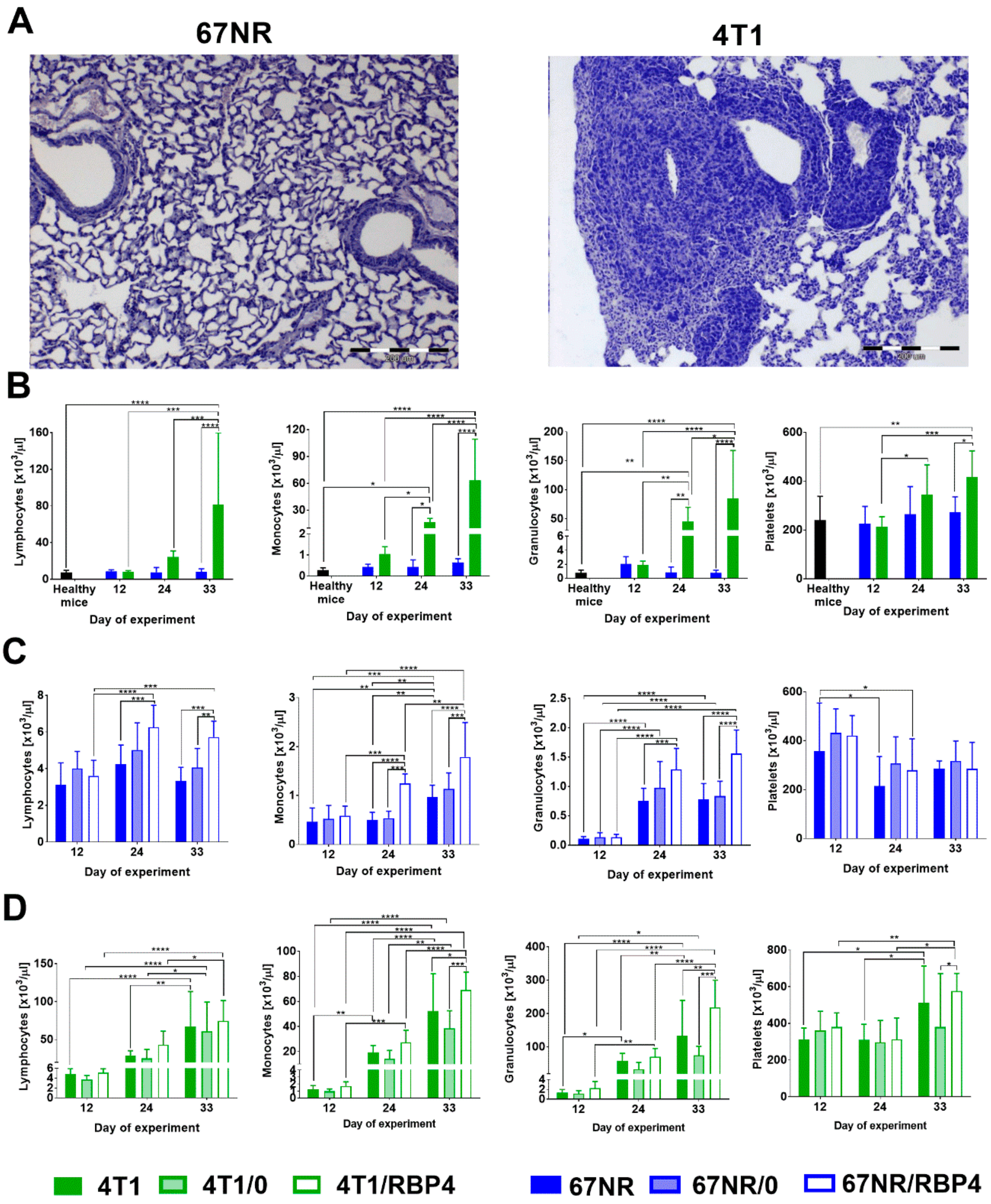

Figure S1. Selected blood morphological parameters of mice bearing 67NR and 4T1 tumors. (**A**) Lung microphotographs from mice bearing 67NR/0 and 4T1/0 cells. (**B**) comparison of 67NR and 4T1

bearing mice. (**C**) Mice bearing 67NR, 67NR/0 and 67NR/RBP4 cells. (**D**) Mice bearing 4T1, 4T1/0, 4T1/RBP4 cells. Count of lymphocytes; monocytes; granulocytes; platelets. Blood from healthy mice as a control was analyzed. Number of mice 6–9/group. Statistical analysis: Tukey's multiple comparison test.* $p < 0.05$, ** $p < 0.01$, *** $p < 0.001$, **** $p < 0.0001$.

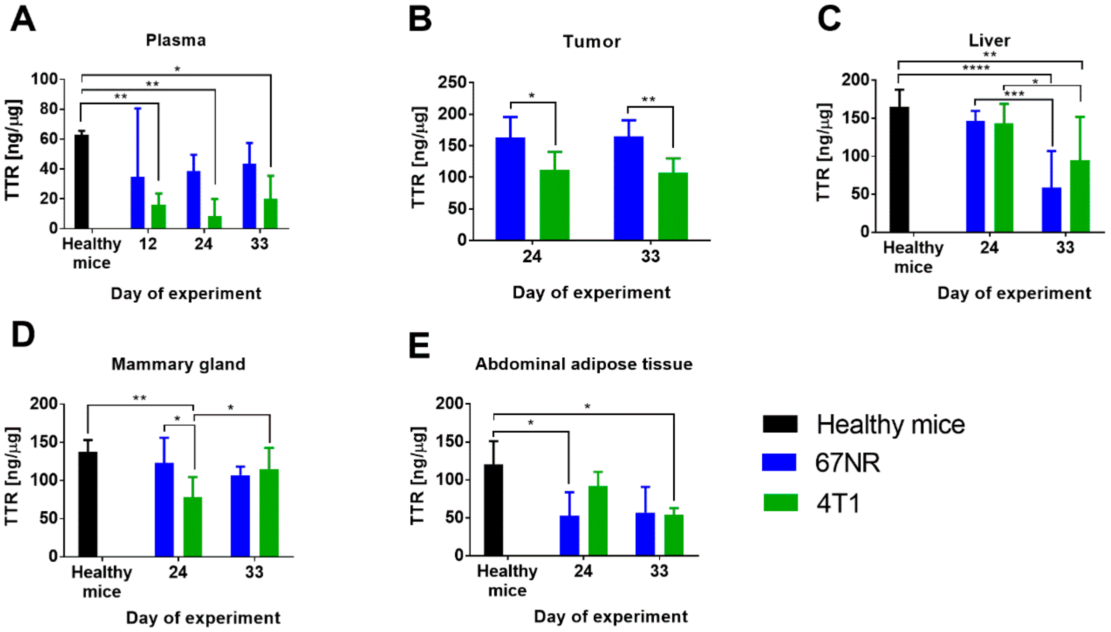

Figure S2. The level of TTR protein in plasma and various tissues from mice bearing nonmetastatic 67NR and metastatic 4T1 mammary gland cancer cells (young mice). (**A**) Plasma, (**B**) tumor tissue, (**C**) liver, (**D**) mammary gland and e abdominal adipose tissue from. Data presented as mean ± SD. Number of mice per group: 6–9. Statistical analysis: Tukey's multiple comparisons test * $p < 0.05$, ** $p < 0.01$, *** $p < 0.001$, **** $p < 0.0001$.

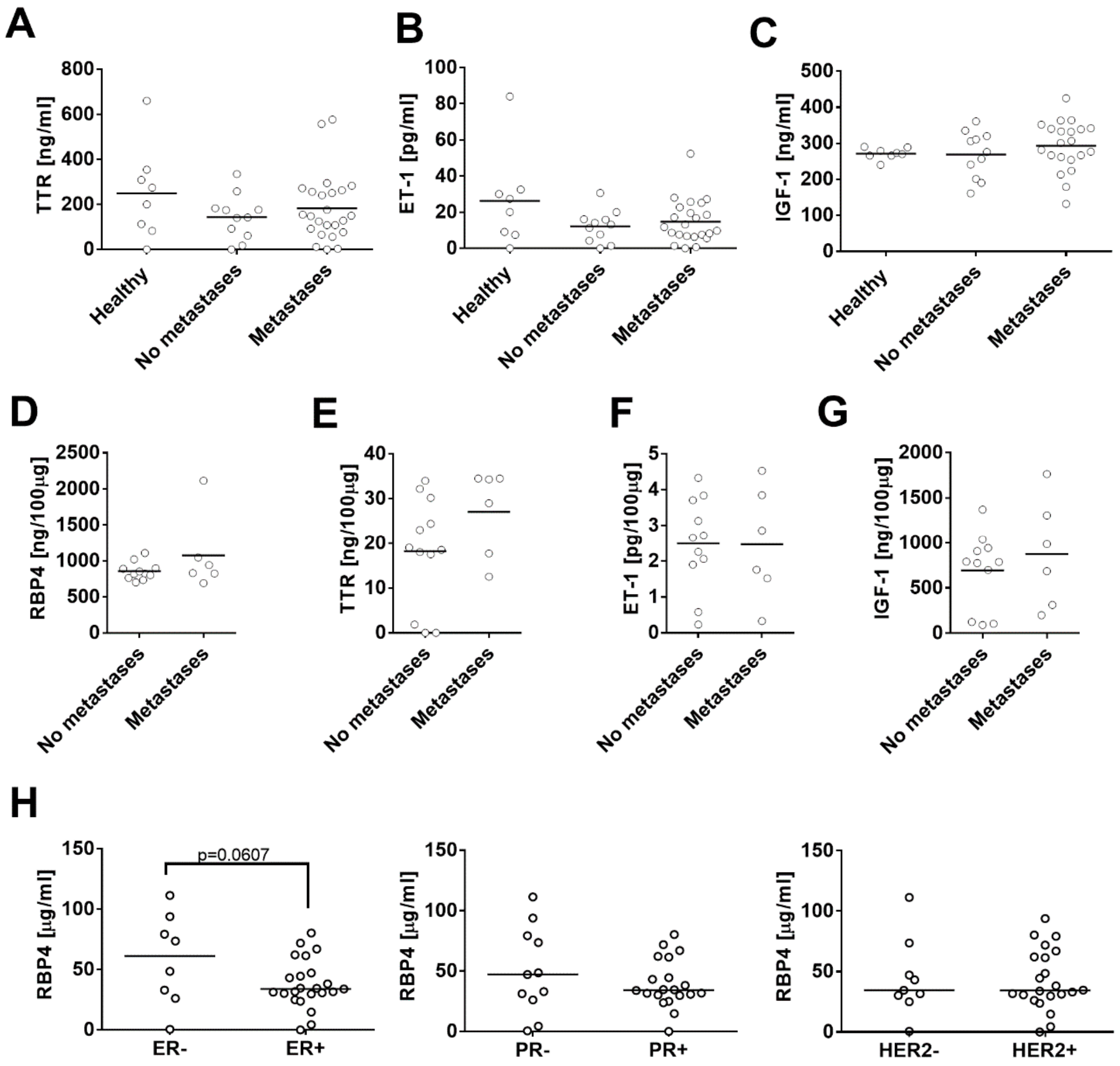

Figure S3. Patients plasma and tumor tissue levels of: RBP4 (tumor tissue), TTR, ET-1 and IGF-1. Plasma level of (**A**) TTR, (**B**) Endothelin-1 (ET-1), (**C**) IGF-1. Tumor tissue level of (**D**) RBP4, (**E**) TTR, (**F**) ET-1, (**G**) IGF-1. (**H**) Plasma level of RBP4 according to receptors status.

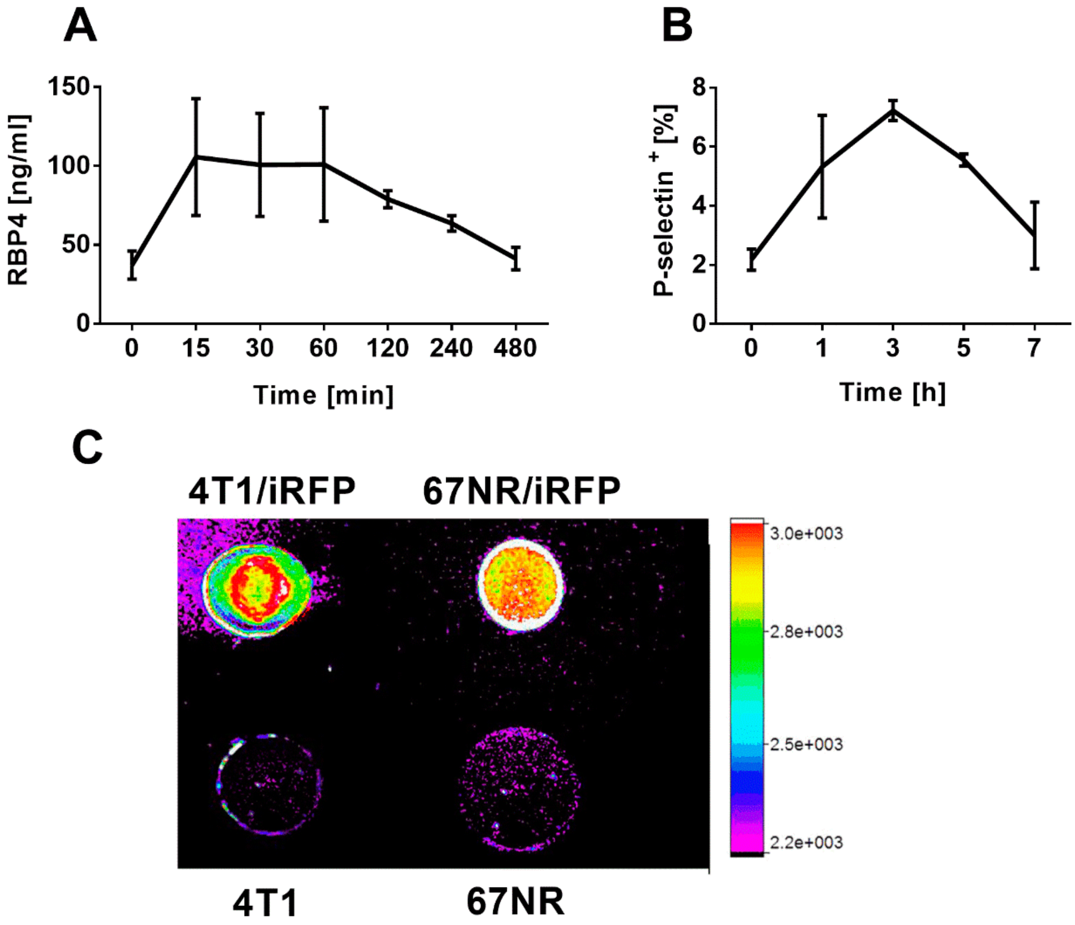

Figure S4. Kinetics of plasma level of RBP4 and in vitro kinetics of endothelial cells activation after incubation with RBP4. (**A**) Kinetics of plasma level of RBP4 after intravenous injection (500 ng/mouse). Three mice were injected with RBP4 and blood was collected at indicated time-points. RBP4 level was analyzed using an ELISA kit. (**B**) P-selectin expression on BALB-5011 endothelial cells cultured in vitro with 200 ng/mL of RBP4. BALB-5011 primary lung microvascular endothelium cells cultured in Complete Mouse Endothelial Cell Medium Kit (Cell Biologics, Chicago, USA) were exposed to 200 ng/ml of RBP4, next the cells were collected, incubated with PE anti-P-selectin 62P antibody (BD Biosciences, San Jose, USA) and analyzed ($N = 4$) in a BD Fortessa cytometer using the Diva software (Becton Dickinson, East Rutherford, USA). (**C**) The efficacy of iRFP670 transfection in mouse mammary gland carcinoma cells. The cell suspension from in vitro culture at a density of 1×10^6 in a 0.5 mL volume was applied to the plate and the fluorescence was read using InVivo MS FX Pro (Carestream Health INC., USA).

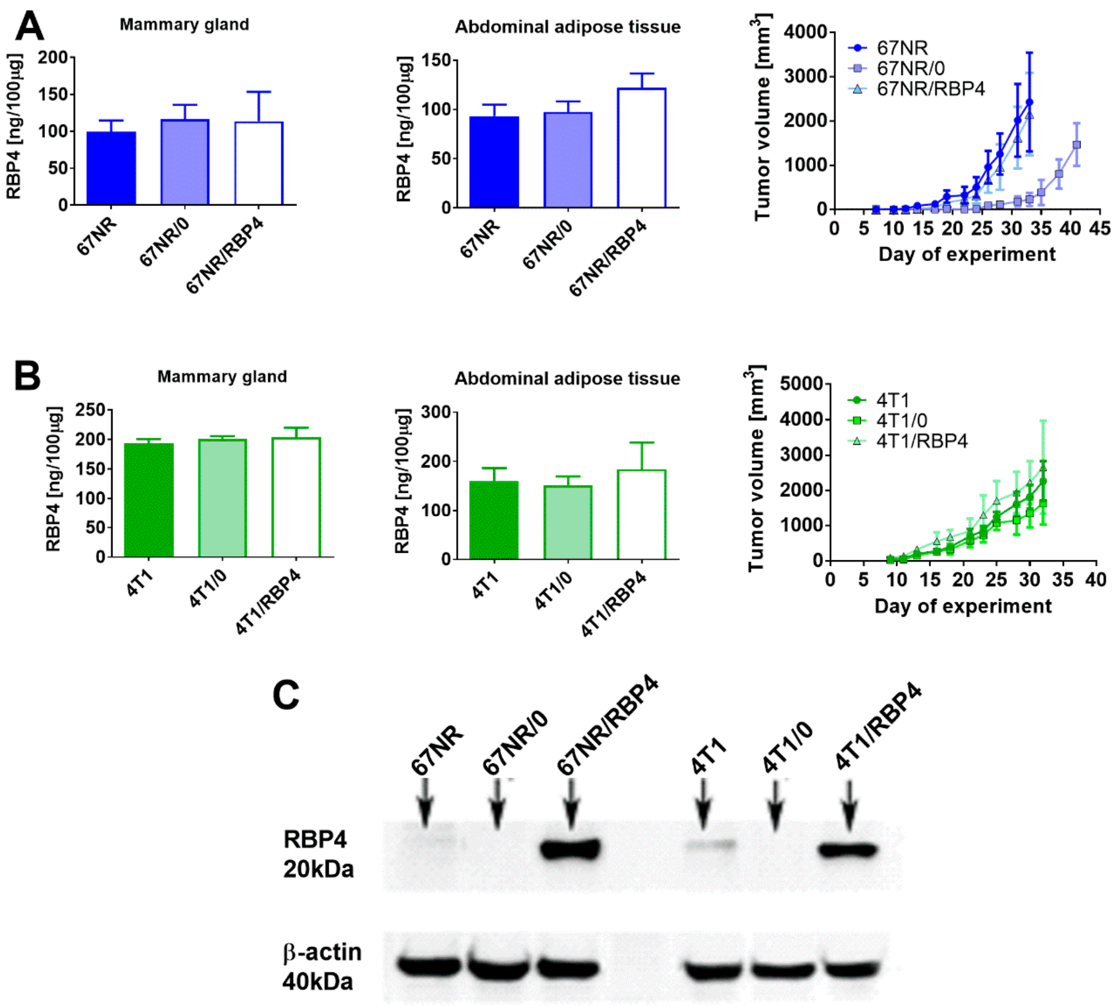

Figure S5. The effect of RBP4 overexpression on kinetics of 67NR/RBP4 and 4T1/RBP4 tumor growth as well as the level of RBP4 in mammary gland and abdominal adipose tissue ((**A**) and (**B**)). (**C**) Western blot analysis of cells from in vitro culture used for induction of 67NR/RBP4 and 4T1/RBP4 tumors.

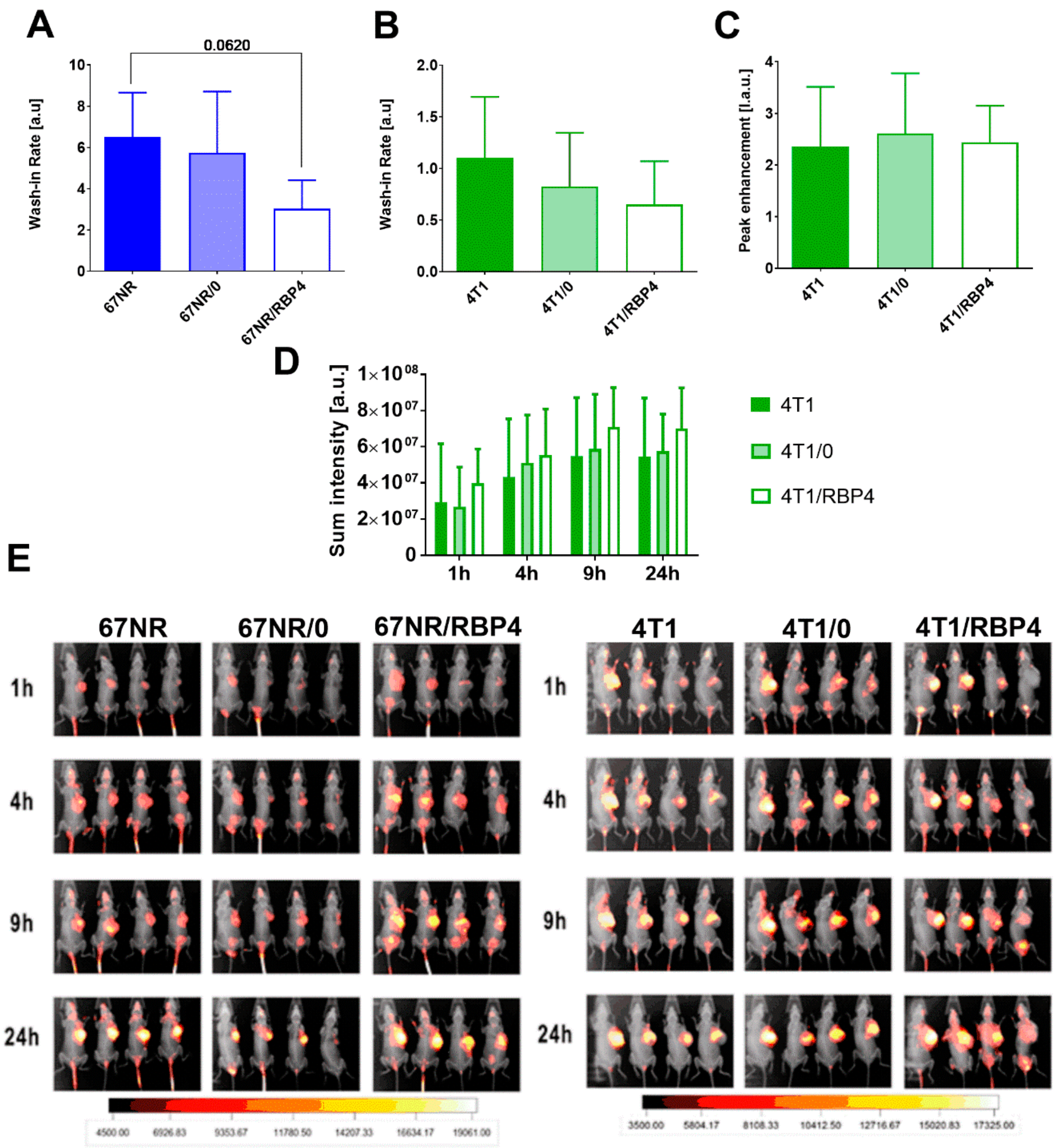

Figure S6. The effect of RBP4 overexpression on angiogenesis of 67NR and 4T1 tumors. Wash in rate parameter estimated for (**A**) 67NR/RBP4 and (**B**) 4T1/RBP4 tumors and appropriate wild type and empty vector controls. (**C**) Peak enhancement in tumor tissue of mice bearing 4T/RBP4 tumors. (**D**) Blood vessel permeability in 4T1/RBP4 tumors and e pictures showing fluorescence measurements (on X-ray images) in all mice in which blood vessel permeability was estimated after 1, 4, 9 and 24 h after contrast agent injection.

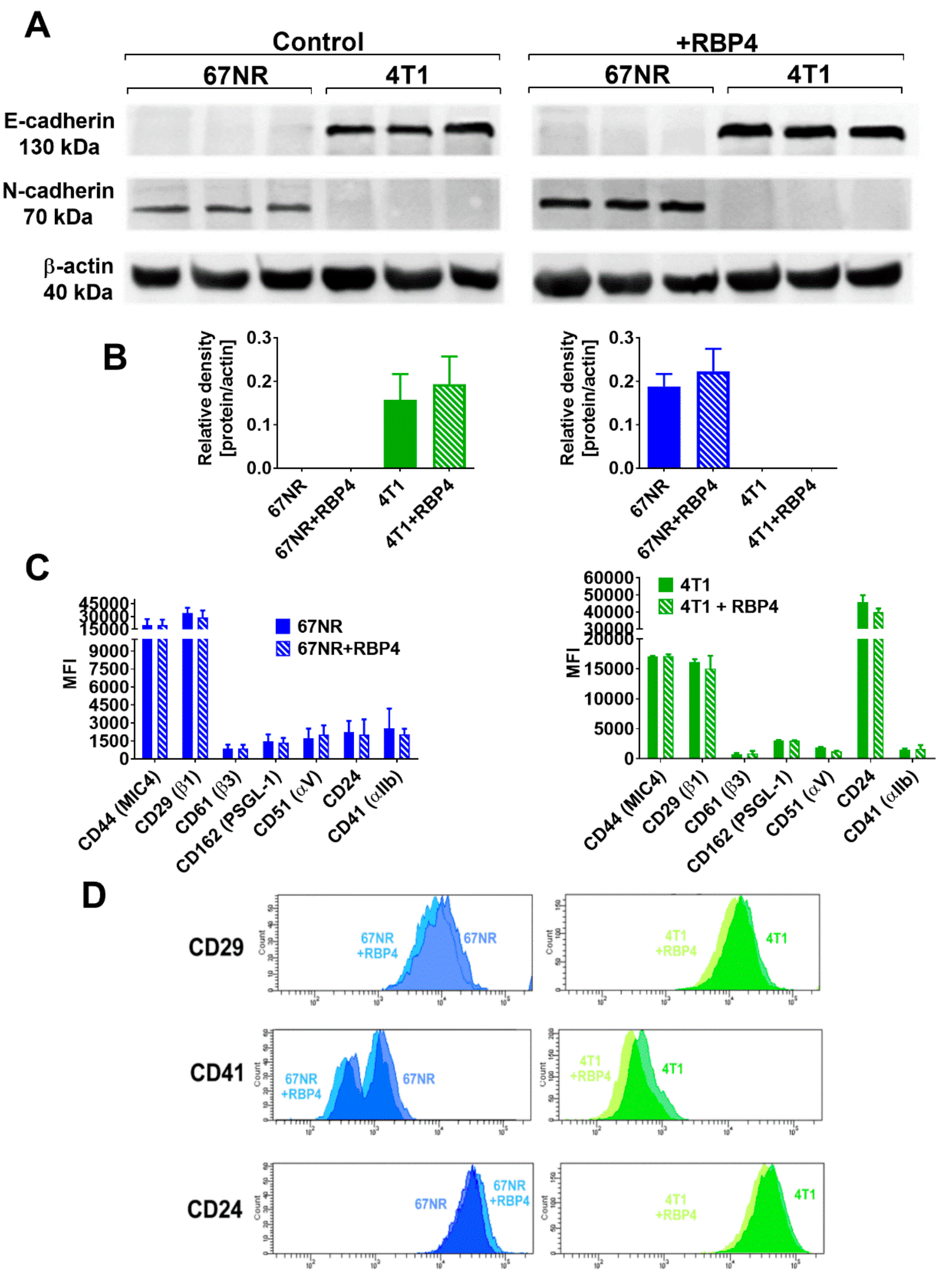

Figure S7. Expression of surface molecules after 24 hours in vitro incubation of 67NR and 4T1 cells with RBP4 protein at a concentration of 200 ng/mL (**A**) Expression of E-cadherin and N-cadherin – representative blot, (**B**) densitometric analysis normalized to expression of β-actin. (**C**) Mean fluorescence intensity for analyzed by flow cytometry adhesion molecules, (**D**) representative histograms. Statistical analysis was performed using the t test.

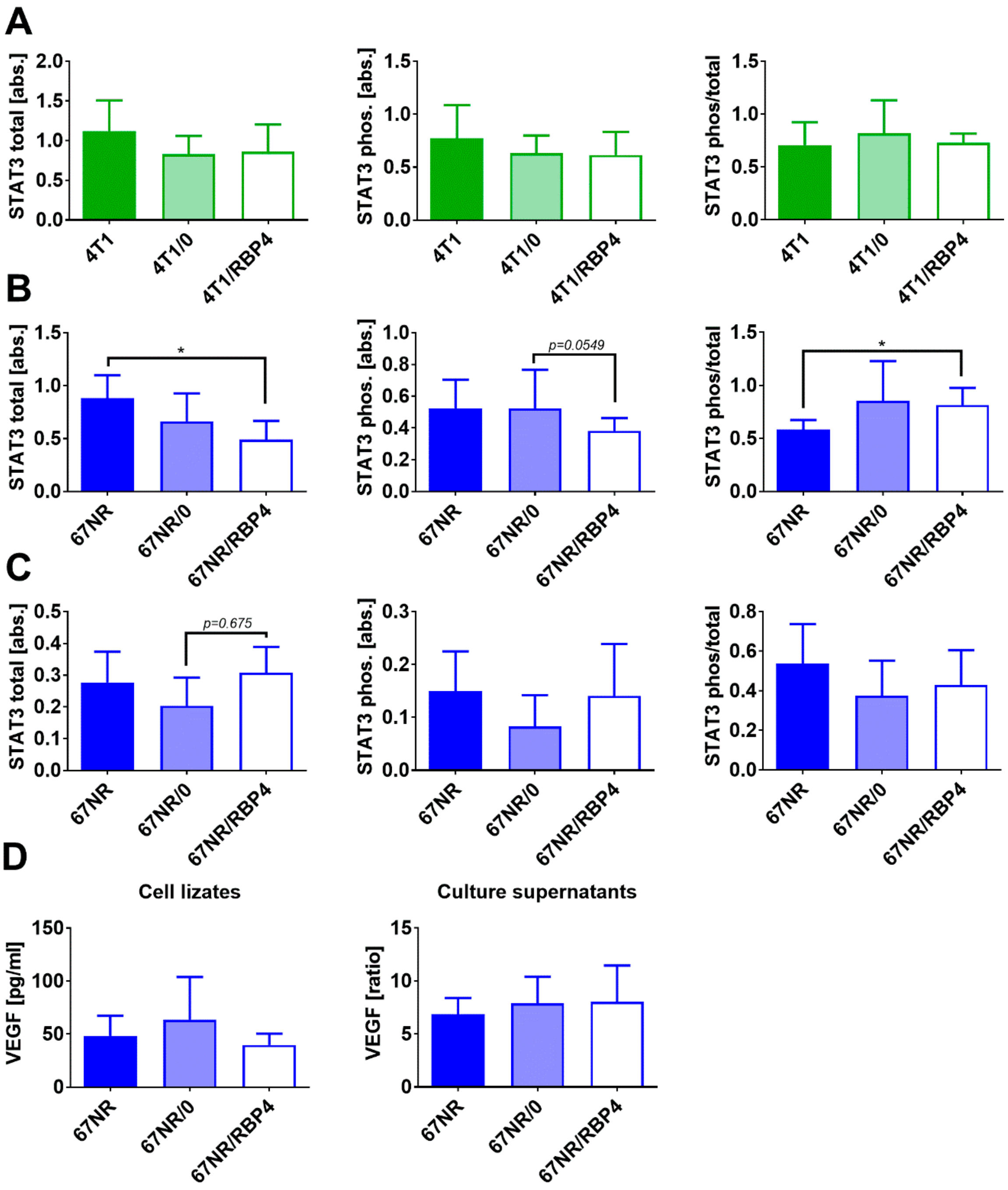

Figure S8. STAT3 phosphorylation and VEGF expression in cell lines with overexpression of RBP4. STAT3 phosphorylation status in tumor lysates of (**A**) 4T1/RBP4 and (**B**) 67NR/RBP4 cells and appropriate controls (N = 6–9). (**C**) STAT3 phosphorylation status in 67NR/RBP4 cells from *in vitro* culture (N = 5–6). (**D**) VEGF level in cell lysates and cell culture medium (VEGF ratio means VEGF level in pg/mL normalized to the protein level of cell lysates. Calculated to avoid differences in cell culture density impact on the level of VEGF release to the culture medium) (N = 7). Data presented as mean with SD. Statistical analysis: Dunn's multiple comparisons test. * $p < 0.05$.

© 2020 by the authors. Licensee MDPI, Basel, Switzerland. This article is an open access article distributed under the terms and conditions of the Creative Commons Attribution (CC BY) license (http://creativecommons.org/licenses/by/4.0/).
